# Development of computational design for reliable prediction of dielectric strengths of perfluorocarbon compounds

**DOI:** 10.1038/s41598-022-10946-x

**Published:** 2022-04-29

**Authors:** Joonho Jang, Ku Hyun Jung, Ki Chul Kim

**Affiliations:** 1 Division of Chemical Engineering, Konkuk University, Seoul, 05029 Republic of Korea; 2 Computational Materials Design Laboratory, Department of Chemical Engineering, Konkuk University, Seoul, 05029 Republic of Korea

**Keywords:** Electrical and electronic engineering, Applied mathematics, Scientific data

## Abstract

The development of robust computational protocols capable of accurately predicting the dielectric strengths of eco-friendly insulating gas candidates is crucial; however, it lacks relevant efforts significantly. Consequently, a series of computational protocols are employed in this study to enable the computational prediction of polarizability and ionization energy of eco-friendly, perfluorinated carbon-based candidates, followed by the equation-based prediction of their dielectric strength. The validation process associated with the prediction of the afore-mentioned variables for selected datasets confirms the suitability of the B3LYP-based prediction protocol for reproducing experimental values. Subsequently, the validation of dielectric strength prediction outlines the following three conclusions. (1) The referenced equation adopted from a previous study is incapable of predicting the dielectric strengths of 137 organic compounds present in our database. (2) Parameterization of the coefficients in the referenced equation leads to the accurate prediction of the dielectric strengths. (3) Incorporation of a novel variable, viz*.* molecular weight, into the referenced equation combined with the parameterization of the coefficients leads to a robust protocol capable of predicting dielectric strengths with high efficiencies even with a significantly smaller fitting dataset. This implies the development of a comprehensive solution capable of accurately predicting the dielectric strengths of a substantially large dataset.

## Introduction

Sulfur hexafluoride (SF_6_) is a synthetic fluorinated compound with an extremely stable chemical structure^1,2^. Owing to its excellent insulation performance, SF_6_ has been widely used as an insulating gas in high-voltage transmission systems; the advantages of SF_6_ over liquid and solid phase materials include its minimal weight, cost-effectiveness, facile manufacturing process, and recyclability^3^. Despite its utility, SF_6_ has been criticized for its negative impact on the environment^4^. For instance, SF_6_ is a well-known greenhouse gas capable of trapping infrared radiations 22,800 times more effectively than CO_2_^5^. As a result, significant efforts have been made to identify potential alternatives to SF_6_ to address serious environmental issues related to its usage^6–11^. Charton et al. and Cooper et al. revealed that dichlorodifluoromethane (CCl_2_F_2_) and carbon tetrafluoride (CF_4_) demonstrated relatively high dielectric strengths in comparison to N_2_^6,11^. Additionally, Xiao et al. explored the effect of metal nanoparticles, such as Cu, Al, and Fe, on the insulation property of trifluoroiodomethane (CF_3_I) and demonstrated that an increase in the electronic conductivity induced by Cu and Al decreased the insulation strength of iodomethane (CH_3_I), further decreasing the breakdown voltage^7^. Moreover, Pagliaro et al. discovered that perfluorinated ketones, including 1,1,1,2,2,4,5,5,5-Nonafluoro-4-(trifluoromethyl)-3-pentanone (NOVEC 1230), could play a crucial role in the development of a novel eco-friendly insulating gas^9^.

With the high level of interest in alternative candidates represented by eco-friendly, perfluorinated carbon-based insulating gases, dielectric strength has become a crucial parameter for determining the insulation performance of these gases^12^. Electrical breakdown occurs within a gas when the dielectric strength of the gas is exceeded. Under a certain amount of electric field, electrons are accelerated by the electric stress applying a force on them, releasing free electrons. These free electrons collide with the gas molecules and part of the kinetic energy of the electrons is transmitted to the molecule, which may cause the ionization and electrical conduction. This is done deliberately in low pressure discharges, such as in fluorescent light. Consequently, the dielectric strengths of numerous perfluorinated organic compounds were experimentally determined to construct a complete database with insulation properties. In this context, Wang et al. evaluated the insulation performance for a selected set of environmentally friendly insulating gas alternatives based on experimental data^12^. However, despite its suitability for measuring a small set of insulating gases, the experimental technique would be inefficient in the evaluation for a large set of organic compounds. In this regard, supplementing analyses with a computational protocol appears to be highly beneficial to facilitate the successful completion of a large-scale database, although relevant efforts are still in their infancy. For instance, Zhang et al. employed the density functional theory (DFT) modeling approach in conjunction with a correlating equation to determine the dielectric strengths for a selected set of organic compounds^13^.

In this study, a series of organic compounds are introduced to develop a robust computational protocol capable of accurately predicting their dielectric strengths based on two fundamental variables, viz*.* polarizability and ionization energy. The DFT-based modeling approach is strategically designed to ensure the accurate prediction of these fundamental variables. Furthermore, they are subsequently utilized to develop a mathematically formulated protocol for the prediction of dielectric strength. The findings demonstrate that all the levels of theory are sufficiently robust for the reliable prediction of the afore-mentioned variables. It is further revealed that the accurate prediction of the dielectric strength for a broad array of organic compounds would be accomplished by incorporating a novel variable, viz*.* molecular weight, into the mathematical formula.

## Methodology

### DFT-assisted prediction of dielectric strength

The dielectric strength of an organic compound relies on multiple variables, such as polarizability, ionization energy, electron affinity, and molecular mass. However, a couple of core variables, viz*.* polarizability and ionization energy, function as the decisive factors for dielectric strength despite the contribution of each of the afore-mentioned variables^13^. Consequently, the DFT modeling approach was employed to determine the polarizability and ionization energy values for a selected set of organic compounds. All the DFT calculations were performed using GAUSSIAN 16 package with four distinct functional types [Becke-3–Lee–Yang–Parr (B3LYP), pure functional of Perdew, Burke and Ernzerhof (PBE1PBE), hybrid functional of Truhlar and Zhao (M062X), and Minnesota density functional (M11)] and 6–311 + G(d, p) basis set^14–18^. Notably, the DFT levels of theory were selected to guarantee a certain level of accuracy (i.e., hybrid-GGA and hybrid-meta-GGA) under the condition of computationally reasonable expense. This guideline would assist us to easily identify an optimal DFT levels of theory for the prediction of key variables. Also, it is notable that organic compounds of our interest require the addition of the polarization and diffuse functions to the minimal basis sets due to the following reasons. (i) *Polarization function*: The molecules have multiple chemical bonds with soft structures. (ii) *Diffuse function*: One of the key variables, ionization energy, is predicted by the DFT calculations of both the neutral and cationic systems. (iii) The diffuse functions of heavy elements need to be preferentially employed considering the balance between the computational accuracy and efficiency. Based on all these, 6–311 + G(d,p) basis set would be the best choice for this study. Subsequently, the DFT-calculated polarizability and ionization energy values were used to predict the dielectric strengths of the organic compounds based on the empirical correlation between the dielectric strength and the two core variables reported previously^13^. More precisely, the correlation can be expressed as follows:

1 $$E_{cal} = 0.0012{\upalpha }^{1.181} (\varepsilon_{i}^{a} )^{1.768}$$

Here, ${E}_{cal}$, $\mathrm{\alpha }$, and ${{\varepsilon }_{i}}^{a}$ denote the dielectric strength, polarizability, and adiabatic ionization energy of an organic compound, respectively. Additionally, the values 0.0012, 1.181, and 1.768 in the equation are the parameterized coefficients for the accurate prediction of organic compounds introduced in a previous study^13^. Notably, the global minimum structure of each organic compound was identified by considering all available molecular configurations before the computational characterization of the core variables and properties.

The correlating equation mentioned above was further improved using two distinct approaches to reliably predict the dielectric strengths for a broader array of organic compounds. In the first approach, the coefficients (*x*, *y*, and *z*) of the equation were further parameterized for the broader array of organic compounds, based on the following equation:

2 $${E}_{cal}={x\mathrm{\alpha }}^{y}{{{(\varepsilon }_{i}}^{a})}^{z}$$

In the second approach, a new variable, viz*.* molecular weight (M_w_), was introduced into the original equation and the resultant equation is defined as follows:

3 $${E}_{cal}={x\mathrm{\alpha }}^{y}{{{(\varepsilon }_{i}}^{a})}^{z}{{M}_{w}}^{m}$$

Notably, the molecular weight of each organic compound may significantly affect its dielectric strength. Additionally, the coefficients (*x*, *y*, *z*, and *m*) of Eq. (3) were further parameterized to include the broader set of organic compounds.

### Materials database

A preliminary search revealed that the list of organic compounds with experimentally determined values was strongly dependent on the property under investigation, such as polarizability, ionization energy, and dielectric strength. Consequently, three distinct datasets were prepared, based on the desired property. In particular, a total of 54 (Fig. S1), 48 (Fig. S2), and 137 (Fig. S3) organic compounds were selected to compile the lists for the polarizability, ionization energy, and dielectric strength values, respectively, obtained from previous references^19–21^.

## Results and discussion

**Figure 1:**
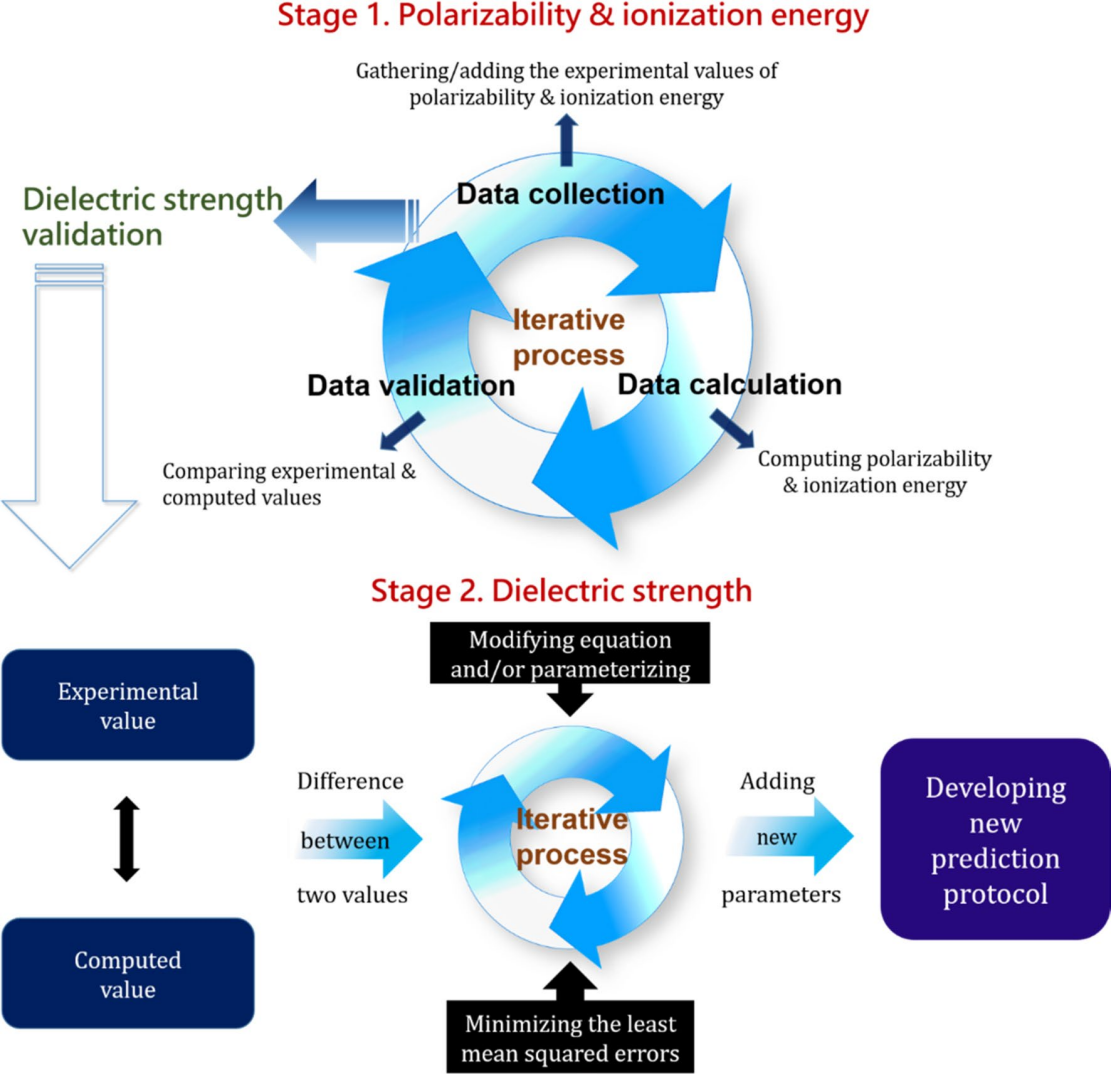
Designed computational protocol. Schematic illustration of developing a computational protocol to predict reliably the dielectric strengths of organic compounds.

Two stages were employed to develop novel computational protocols for predicting the dielectric strengths of organic compounds in the gas phase (Fig. 1). The first stage involved the computation of two fundamental variables, polarizability and ionization energy, for a diverse set of selected organic compounds (Figs. S1 and S2 in the Supporting Information, respectively) using the DFT method with four distinct functional types. The reliability of the computed values was evaluated by comparison with their experimental values to optimize the DFT-assisted computational protocol. The second stage mathematically correlated the two core variables computed in the previous stage using the optimized DFT-based protocol with dielectric strength using an appropriate equation. The primary goal of the second stage was to optimize the correlation through the comparison between the equation-assisted and experimental dielectric strengths for a given set of organic compounds (Fig. S3 in the Supporting Information). Furthermore, a series of equation candidates (Eqs. 1, 2, and 3) were employed to optimize the correlation.

### Stage 1: Validation of computational protocol for polarizability and ionization energy

#### Polarizability

**Figure 2:**
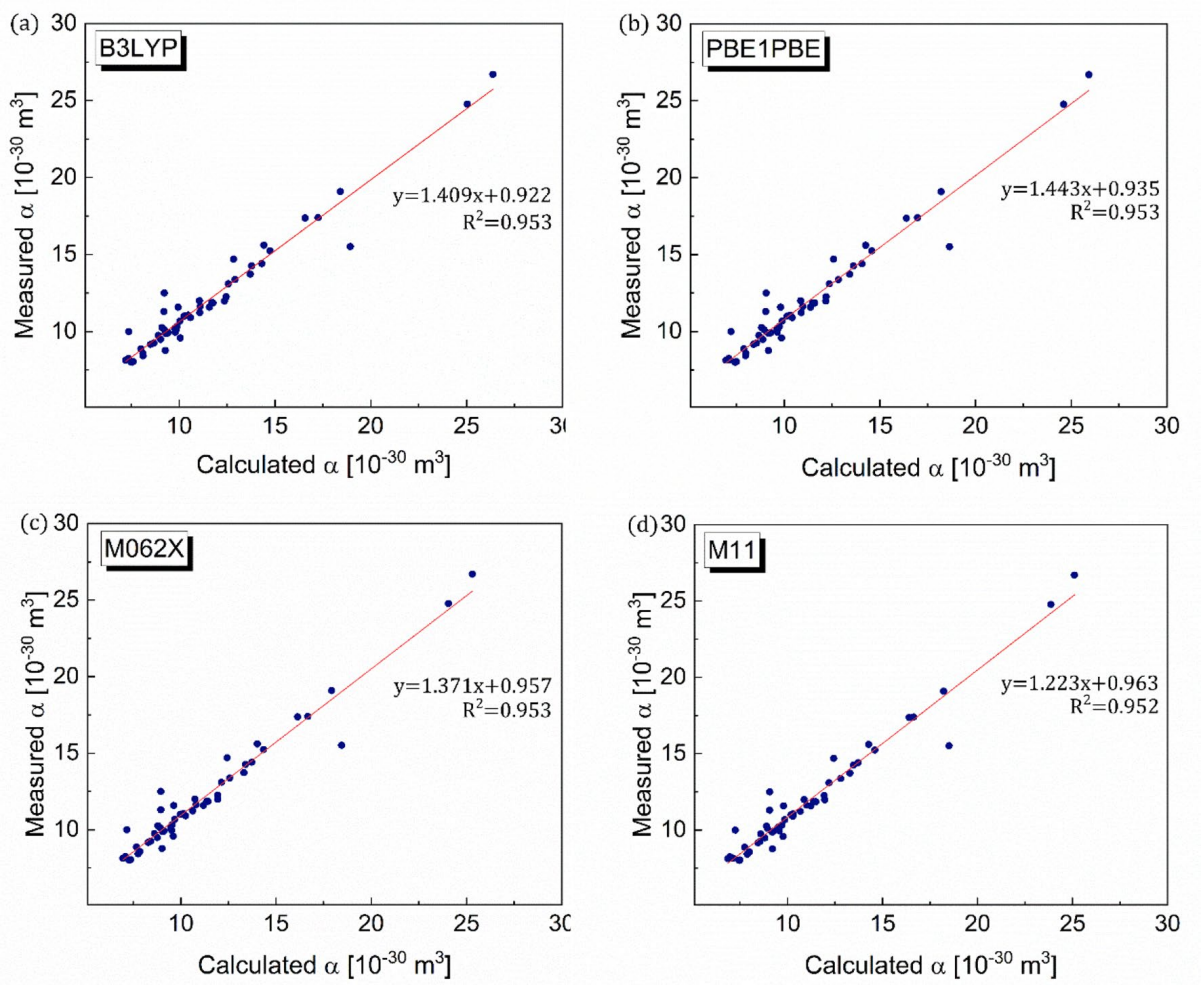
Validation of DFT method for polarizability. Comparisons between DFT-computed and measured polarizability (α) values for four different DFT functionals, viz*.* (**a**) B3LYP, (**b**) PBE1PBE, (**c**) M062X, and (d) M11.

The polarizability values of 54 organic compounds computed via the DFT modeling approach with four distinct DFT functional types, viz*.* B3LYP, PBE1PBE, M062X, and M11, are shown in Fig. 2. The computed values correspond to their experimental values via trend lines, *y* = [(~ 1.22–1.44)]*x* + (~ 0.92–0.96), which are close to *y* = *x*, with the least-squares of 0.952–0.953, irrespective of the DFT functional type. Additionally, the least-squares with respect to *y* = *x* indicate how close the DFT-calculated values are to their experimental values. The analyzed root-mean-square deviation (RMSD) values with respect to *y* = *x* imply that all DFT functional types reliably predict the polarizability values of the organic compounds with acceptable degrees of error ~ 7.46–8.98, with the B3LYP DFT functional exhibiting the lowest error value (Table 1).

**Table 1:** Root-mean-square deviation (RMSD). RMSD values for polarizability, ionization energy, and dielectric strengths with referenced/parameterized equations.

Property		DFT functional	RMSD	Relevant figure
Polarizability		B3LYP	7.46	Figure 2a
		PBE1PBE	8.19	Figure 2b
		M062X	8.98	Figure 2c
		M11	8.57	Figure 2d
Ionization energy		B3LYP	2.02	Figure 3a
		PBE1PBE	1.78	Figure 3b
		M062X	1.30	Figure 3c
		M11	2.52	Figure 3d
Dielectric strength (referenced equation)		B3LYP	9.01	Figure 5a
Dielectric strength (parameterized Eq. 1)	Fitting 30 set	B3LYP	6.09	Figure 6a
	Fitting 60 set		5.95	Figure 6b
	Fitting 90 set		7.28	Figure 6c
	Fitting 137 set		4.13	Figure 6d
Dielectric strength (parameterized Eq. 2)	Fitting 30 set	B3LYP	5.01	Figure 8a
	Fitting 60 set		4.15	Figure 8b
	Fitting 90 set		4.08	Figure 8c
	Fitting 137 set		3.98	Figure 8d

#### Ionization energy

**Figure 3:**
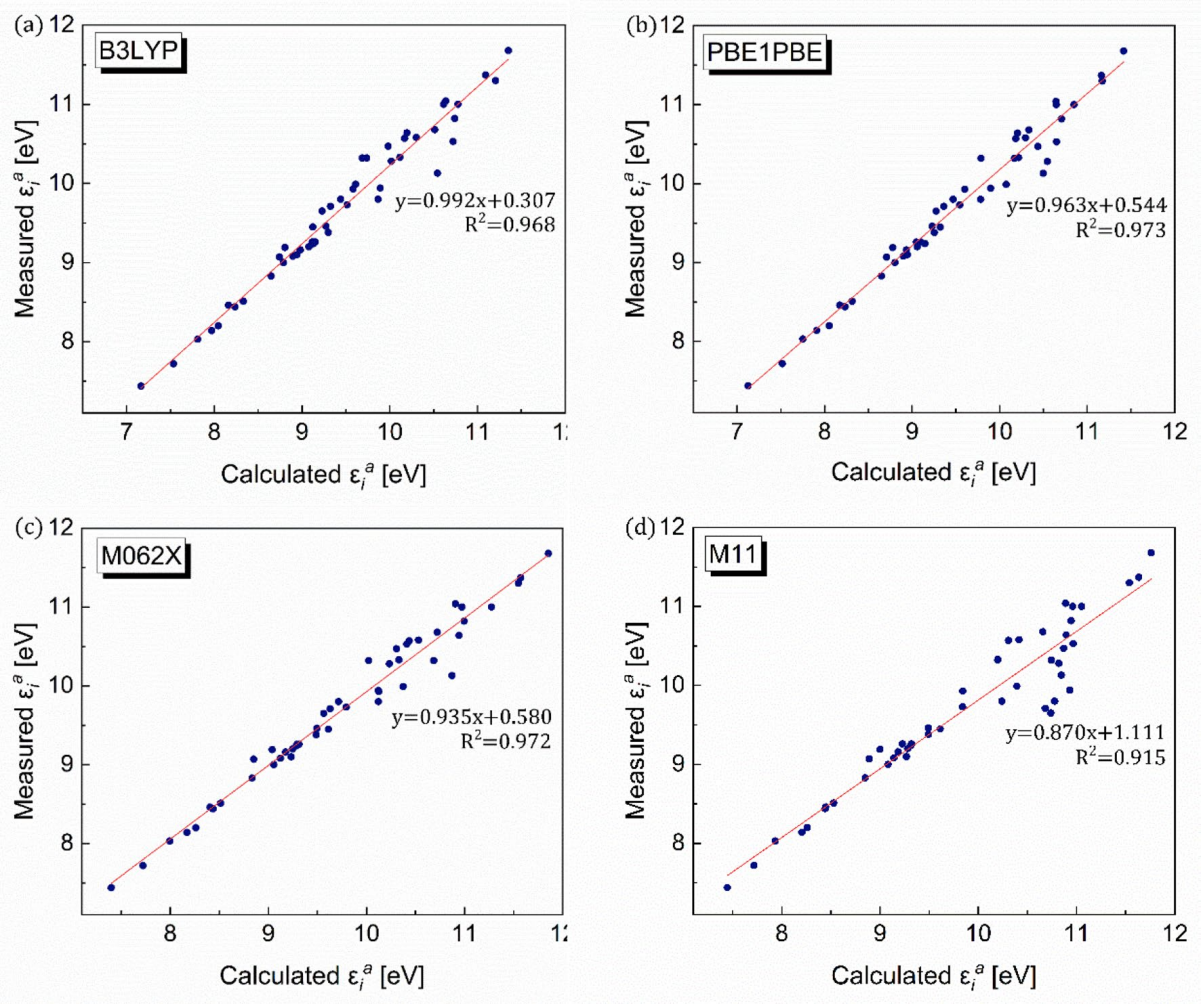
Validation of DFT method for ionization energy. Comparisons between DFT-computed and measured ionization energy (${\varepsilon }_{i}^{a}$) values for four different DFT functionals, viz*.* (**a**) B3LYP, (**b**) PBE1PBE, (**c**) M062X, and (**d**) M11.

The same logic was used to compute the ionization energies of 48 organic compounds using the four DFT functional types employed previously for polarizability validation. Notably, the dataset of organic compounds used to validate the ionization energy is not necessarily identical to that used for the validation of polarizability, primarily due to the potential difference in the availability of experimental information. Likewise, regardless of the DFT functional type, the computed ionization energies agree well with their experimental values, exhibiting trend lines close to *y* = *x* (Fig. 3). B3LYP-computed values, in particular, agree well with their experimental values, exhibiting the trend line closest to *y* = *x* (Fig. 3), with the slope corresponding to almost unity and the y-intercept approaching zero. This observation is further strengthened by the exceptionally low RMSD values of 1.30–2.52 (Table 1).

#### Error distributions of polarizability and ionization energy

**Figure 4:**
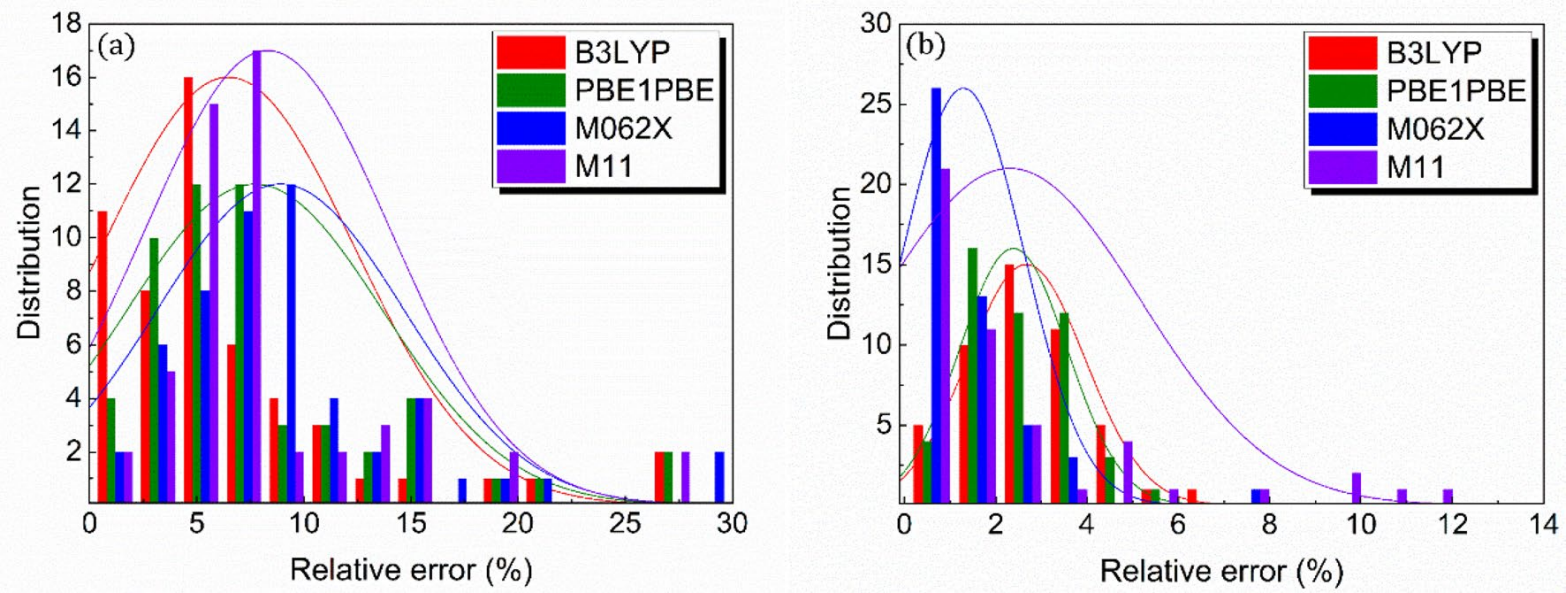
Relative errors for polarizability and ionization energy. Errors of the DFT-computed values relative to their experimental values for (**a**) polarizability and (**b**) ionization energy.

The core variables were further explored through the analyses of the distributions of organic compounds in terms of the errors associated with the DFT-computed values relative to the experimental ones (Fig. 4). For polarizability, in particular, averaged relative errors (fractions of organic compounds with relative errors of less than 10%) of 6.47% (87.03%), 7.71% (75.93%), 8.91% (72.22%), and 8.31% (75.93%) are highlighted for the organic compounds at B3LYP, PBE1PBE, M062X, and M11 levels of theory, respectively. In particular, the B3LYP-based protocol has a greater distribution (26 out of 54 organic compounds) than any other DFT functional types at relative errors of less than 5%. In comparison, the accuracy of the DFT-calculated ionization energy is unlikely to be affected by the DFT functional type, with relative errors of typically less than 5% for the majority of organic compounds. Additionally, averaged relative errors of 1.29–2.67% for the ionization energy are predicted. The findings, therefore, imply that B3LYP is the optimal DFT functional for accurately predicting both polarizability and ionization energy. Thus, all subsequent analyses are based on the B3LYP-based computation.

### Stage 2: Development of novel computational protocols for dielectric strength

The DFT-predicted variables, viz*.* polarizability and ionization energy, were further combined with a given equation (correlation of dielectric strength with polarizability and ionization energy) to predict the dielectric strength values of organic compounds. The equation used to accomplish this objective is classified as (i) referenced equation and (ii) parameterized equation. The referenced equation was adopted from a correlation applicable to a database of 75 organic compounds with experimental dielectric strengths of 0.445–1.959 relative to the SF_6_ value obtained in a previous study^13^. In contrast, the parameterized equations were further developed through the extension/revision of the referenced equation to describe better correlations between the core variables. Notably, 137 organic compounds were introduced as a new dataset to analyze their dielectric strengths from the B3LYP-computed values of polarizability and ionization energy. Based on the above-discussed reason, the dataset of organic compounds utilized for the validation of dielectric strength is not necessarily identical to those utilized for the validation of polarizability and ionization energy.

#### Referenced equation (Eq. 1)

**Figure 5:**
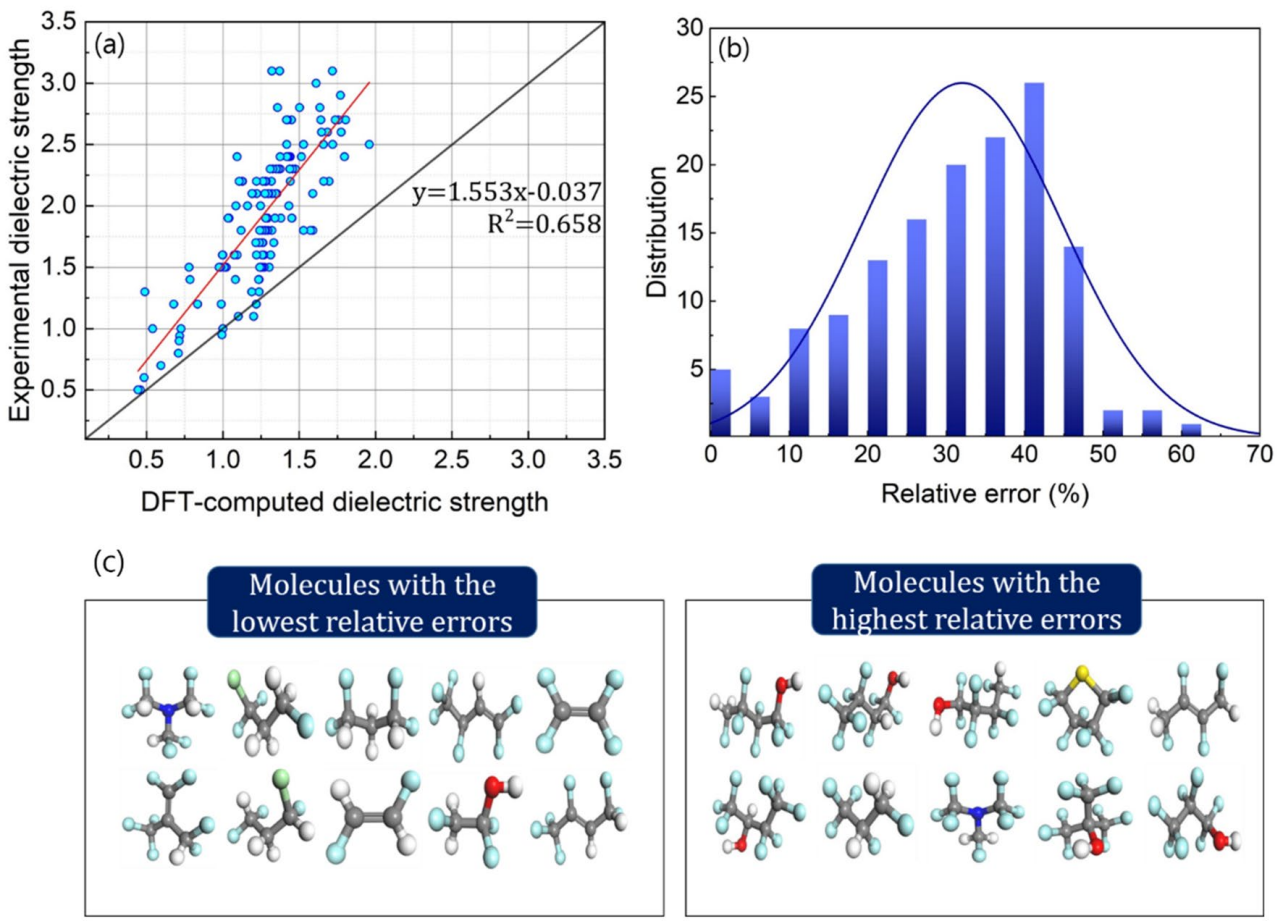
Validation of original computational protocol for dielectric strength. (**a**) Comparisons between the equation-predicted and measured values for the dielectric strength (relative to SF_6_ = 1.0). (**b**) Errors of the equation-predicted dielectric strengths relative to their experimental values. (c) Structures of organic compounds with the lowest and highest relative errors.

Zhang et al. investigated the relationship between dielectric strength, polarizability, and ionization energy for a given set of organic compounds^13^. This correlation was adopted to our dataset to verify the equation's applicability to our organic compounds (Fig. 5). Notably, the dielectric strength of an organic compound is generally reported in relation to that of the representative insulating gas, SF_6_. Interestingly, the computed values frequently underestimate the dielectric strengths of 137 organic compounds in our dataset (Fig. 5a). The computed values follow a trend line of *y* = 1.553*x*−0.037, resulting in an averaged underestimation of approximately 30–40% relative to their experimental values (Fig. 5b). The RMSD of the dielectric strengths predicted for 137 organic compounds is further notated to be 9.01, indicating the referenced equation's limited predictive ability (Table 1).

The structural properties of organic compounds with the lowest and highest errors in the computed dielectric strength were further examined to determine the physical basis for the underestimation of the computed dielectric strength (Fig. 5c). Despite the absence of a discernible difference in the structural property of the two groups with the lowest and highest errors, organic compounds with simpler structures are likely to exhibit lower errors. This may be explained by the fact that the organic compounds used in the previous study to develop the referenced equation have a relatively simple structure^13^.

#### Reparameterized equation (Eq. 2)

**Figure 6:**
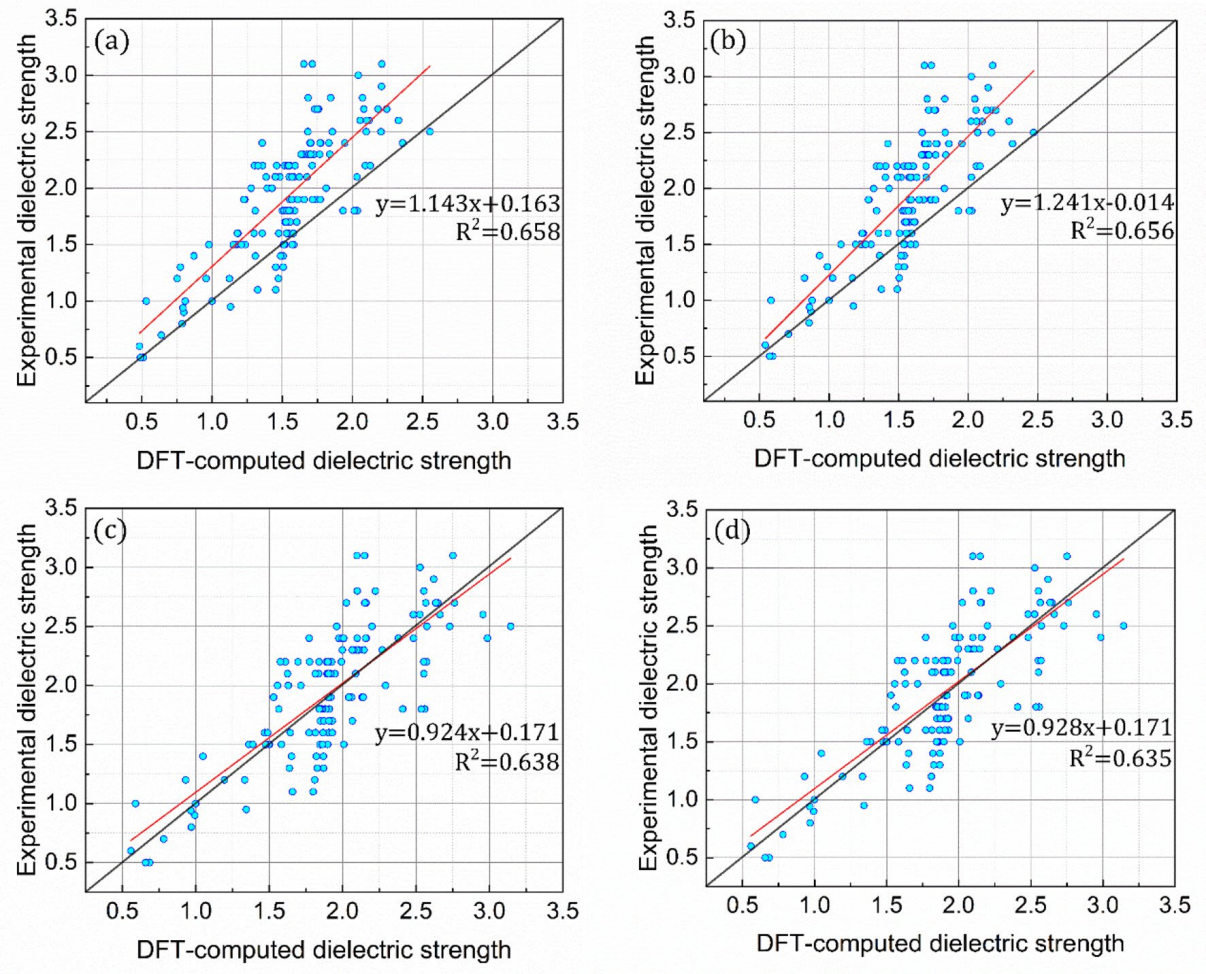
Validation of computational protocol with parameterized coefficients for dielectric strength. Comparisons between the equation-predicted and experimental dielectric strengths for all the organic compounds in Fig. S3 in the Supporting Information. The coefficients of the equations are parameterized for the equation-based prediction of the experimental values of either (**a**) 30, (**b**) 60, (**c**) 90 selected randomly among the organic compounds in Fig. S3 in the Supporting Information, or (**d**) all the compounds. The equations are correlated with polarizability and ionization energy.

**Figure 7:**
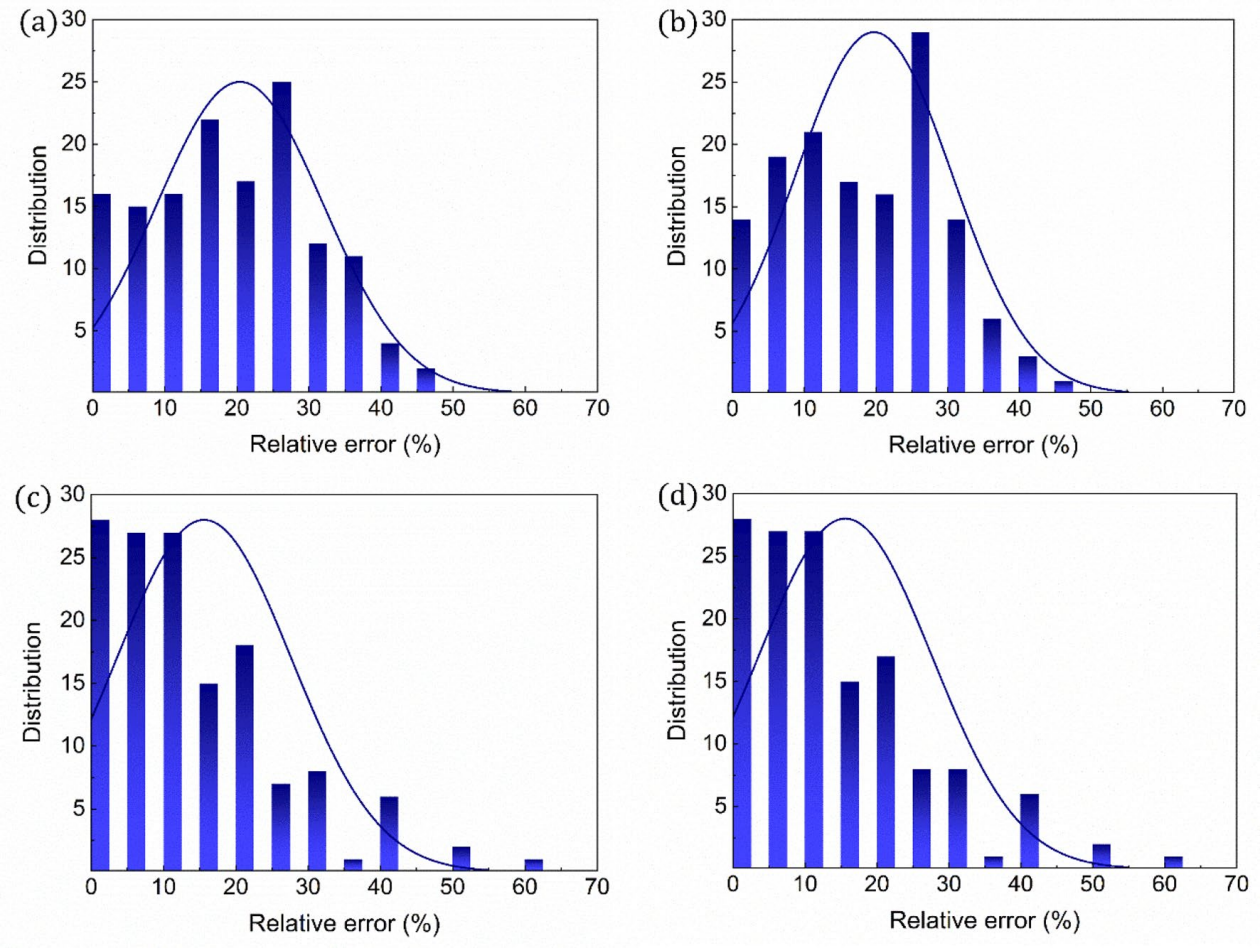
Relative errors for dielectric strength. Errors of the equation-predicted dielectric strengths relative to their experimental values. The coefficients of the equations are parameterized for the equation-based prediction of the experimental values of either (**a**) 30, (**b**) 60, (**c**) 90 or (**d**) all the compounds in Fig. S3 in the Supporting Information. The equations are correlated with polarizability and ionization energy.

The coefficients (*x*, *y*, and *z* in Eq. 2) of the referenced equation were parameterized as the first approach to improve the ability of the referenced equation used for predicting the dielectric strengths of organic compounds. In particular, the coefficients were independently parameterized for the four distinct fitting datasets of randomly selected organic compounds (30, 60, 90, and 137 compounds) to accurately predict their dielectric strengths (Fig. S4). As expected, the parameterized equations (Eq. 2 in conjunction with Table 2) make more accurate predictions in the dielectric strength, with trend lines, *y* = [(~ 0.924–1.241)]*x* + (~ 0.014–0.171) that are close to *y* = *x* (Fig. 6). Moreover, it is unambiguously observed that the equation with the parameterized coefficients for a larger fitting dataset has a superior prediction ability, with the lowest (highest) averaged relative error of 15.49% (20.43%) for the fitting dataset containing 137 (30) organic compounds (Fig. 7). The RMSD values of the dielectric strengths predicted for 137 organic compounds are further predicted to be 6.09, 5.95, 7.28, and 4.13 for the fitting datasets that contain 30, 60, 90, and 137 organic compounds, respectively (Table 1). From these analyses, it is highlighted that 137 organic compounds would be the most suitable dataset for parameterizing the equation coefficients to develop a robust protocol for dielectric strength prediction (Fig. 7).

**Table 2:** Parameterized coefficients. Equation coefficients (*x*, *y*, and *z*) parameterized using either (a) 30, (b) 60, (c) 90 selected randomly among the organic compounds in Fig. S3 in the Supporting Information, or (d) all the compounds. The equations are correlated with polarizability and ionization energy.

${E}_{calc}=x{\left(\alpha \right)}^{y}{\left({\varepsilon }_{i}^{a}\right)}^{z}$	30 of 137	60 of 137	90 of 137	137 of 137	Average
$x$	0.000184	0.000628	0.000459	0.0012	0.000618
$y$	1.362	1.224	1.169	1.356	1.278
$z$	1.490	1.128	1.318	0.760	1.174

#### New equation (Eq. 3)

**Figure 8:**
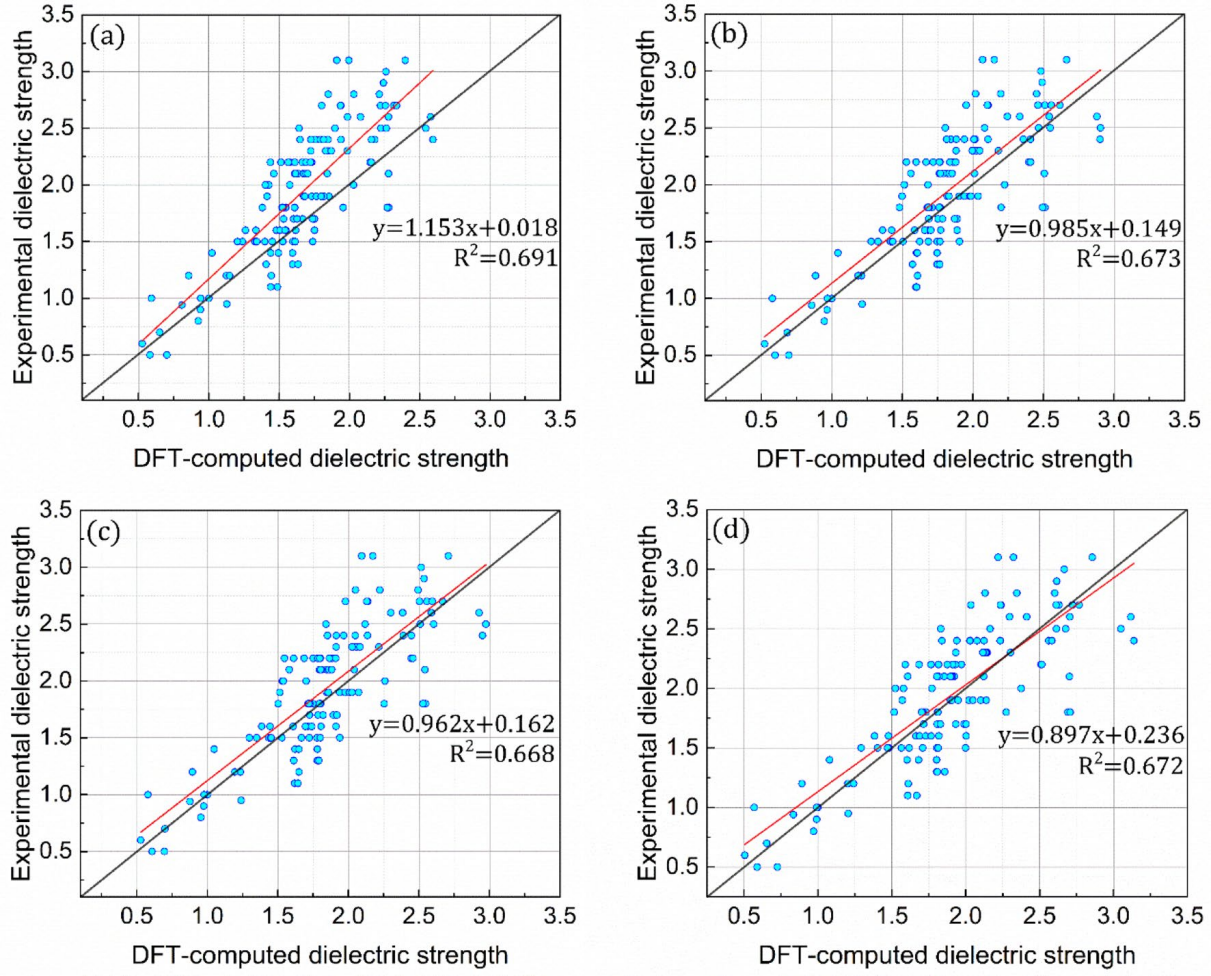
Introduction of a new variable to computational protocol for dielectric strength. Comparisons between the equation-predicted and experimental dielectric strengths for all the organic compounds in Fig. S3 in the Supporting Information. The coefficients of the equations are parameterized for the equation-based prediction of the experimental values of either (**a**) 30, (**b**) 60, (**c**) 90 selected randomly among the organic compounds in Fig. S3 in the Supporting Information, or (**d**) all the compounds. The equations are correlated with polarizability, ionization energy, and molecular weight.

**Figure 9:**
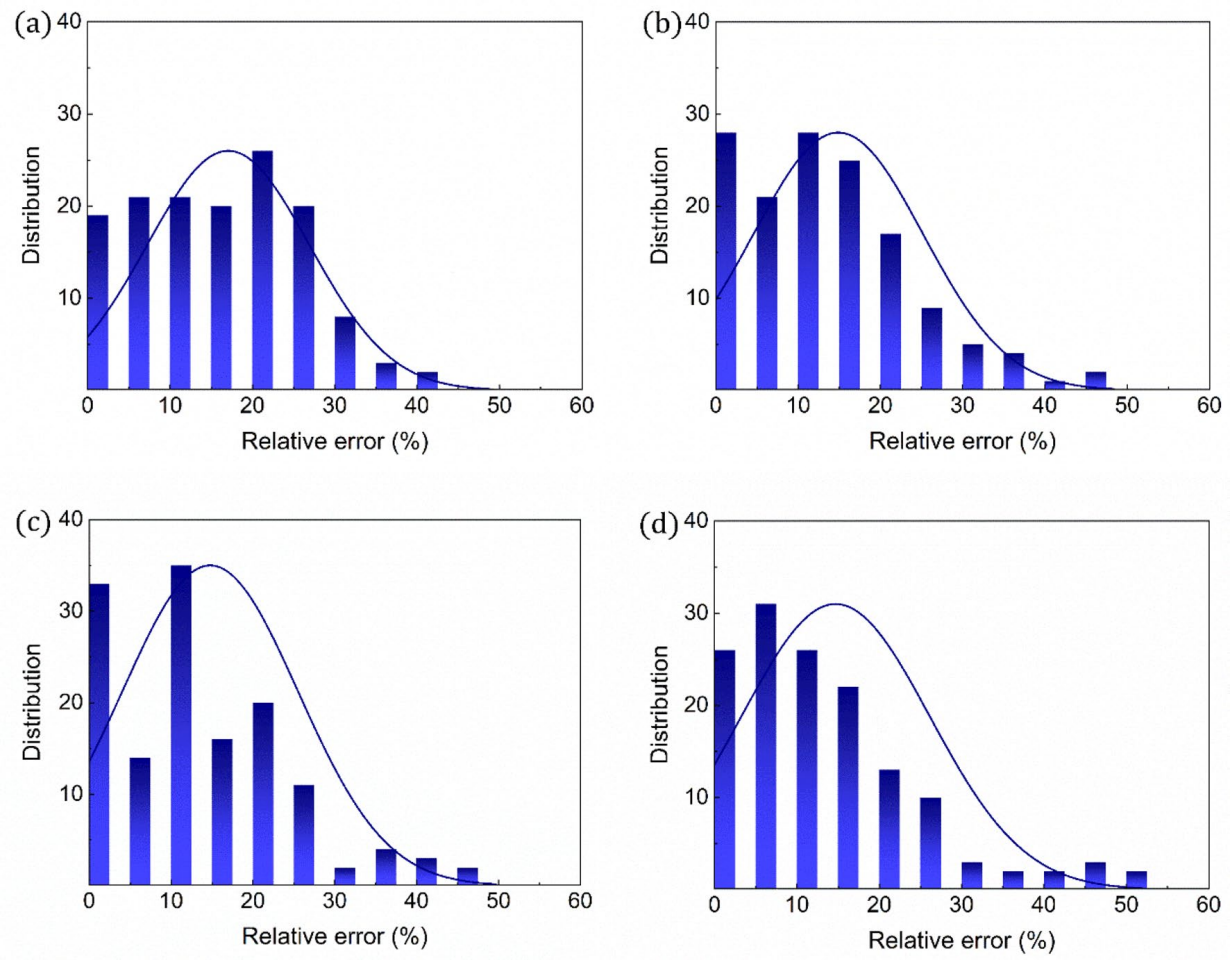
Relative errors for dielectric strength. Errors of the equation-predicted dielectric strengths relative to their experimental values. The coefficients of the equations are parameterized for the equation-based prediction of the experimental values of either (**a**) 30, (**b**) 60, (**c**) 90 or (**d**) all the compounds in Fig. S3 in the Supporting Information. The equations are correlated with polarizability, ionization energy, and molecular weight.

It is noticeable that a molecule with a greater atomic mass has a higher polarizability because a longer distance from its nucleus results in a looser electron, leading to a more easiness in the polarization. Likewise, the ionization energy is often reported as the amount of energy required to ionize the number of atoms or molecules present in one mole, highlighting the intimate relationship between the ionization energy and molecular weight. This implies that the dielectric strength, which is represented by the two key factors, namely polarizability and ionization energy, is expected to be significantly affected by the molecular weight. Therefore, a new variable, viz*.* molecular weight, was introduced to further improve the ability of the above-discussed equation to accurately predict the dielectric strengths of 137 organic compounds. Following a similar logic, the coefficients were independently parameterized for four distinct fitting datasets of randomly selected organic compounds (30, 60, 90, and 137 compounds) to accurately predict their dielectric strengths (Fig. S5). As expected, all the four equations (Eq. 3 in conjunction with Table 3) developed using the distinct fitting datasets outperform the referenced equation in terms of prediction ability, with the trend lines, *y* = [(~ 0.897–1.153)]*x* + (~ 0.018–0.236) (Fig. 8). In particular, the averaged relative errors of 17.07, 14.87, 14.77 and 14.69% are predicted, exhibiting 40, 49, 47, and 57 organic compounds with relative errors of less than 10% for the fitting datasets that contain 30, 60, 90, and 137 organic compounds, respectively (Fig. 9). The RMSD values of 5.01, 4.15, 4.08, and 3.98 for the fitting datasets containing 30, 60, 90, and 137 organic compounds, respectively, are also noteworthy, implying the negligible difference in the RMSD value between the latter three fitting datasets (Table 1).

**Table 3:** Parameterized coefficients. Equation coefficients (*x*, *y*, *z*, and *m*) parameterized using either (a) 30, (b) 60, (c) 90 selected randomly among the organic compounds in Fig. S3 in the Supporting Information, or (d) all the compounds. The equations are correlated with polarizability, ionization energy, and molecular weight.

${E}_{calc}=x{\left(\alpha \right)}^{y}{\left({\varepsilon }_{i}^{a}\right)}^{z}{\left({M}_{w}\right)}^{m}$	30 of 137	60 of 137	90 of 137	137 of 137	Average
$x$	0.0012	0.0012	0.0012	0.0012	0.0012
$y$	0.876	1.083	1.132	1.000	1.022
$z$	0.422	0.517	0.542	0.288	0.442
$m$	0.364	0.263	0.225	0.401	0.313

All of these factors point to an unexpected pivotal evolution in the development of a computational protocol for the reliable and accurate prediction of dielectric strengths. The introduction of molecular weight variable significantly improves the prediction ability, and thus 60 (or even 30) organic compounds are found to be sufficient for the reliable parameterization of the equation coefficients with the RMSD value of 4.15, comparable to the parameterized Eq. (1) with the fitting dataset of 137 compounds. This enables us to further draw a meaningful conclusion on the importance of incorporating the new variable, molecular weight, in the equation, resulting in a reduction in the size of the fitting dataset required for the accurate prediction of the dielectric strengths of 137 organic compounds in the large dataset. This implies that parameterized Eq. (2) guarantees the reliable prediction ability not only for the 137 organic compounds but also for extended datasets with a larger number of organic compounds. Notably, the parameterized Eq. (1) can be designed only using 137 compounds in fitting dataset for the reliable prediction of dielectric strengths. Consequently, the equation does not guarantee that it will accurately predict the dielectric strengths of extended datasets that are larger than the current dataset.

### Structure–property relationship

**Figure 10:**
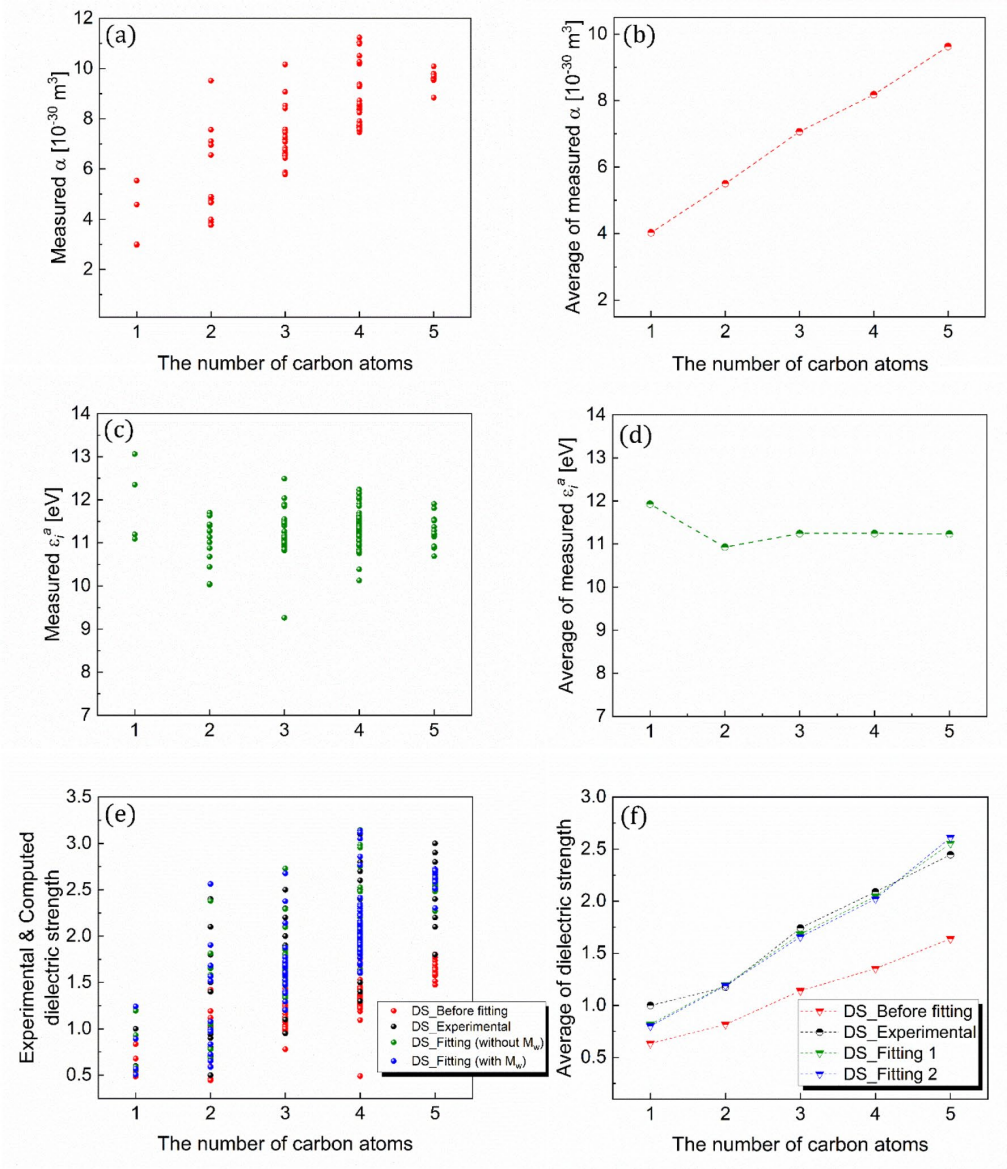
Correlations with structural property. (**a**, **b**) Polarizabilities, (**c**, **d**) ionization energies, and (**e**, **f**) dielectric strengths of the organic compounds correlated with their numbers of carbon atoms.

**Figure 11:**
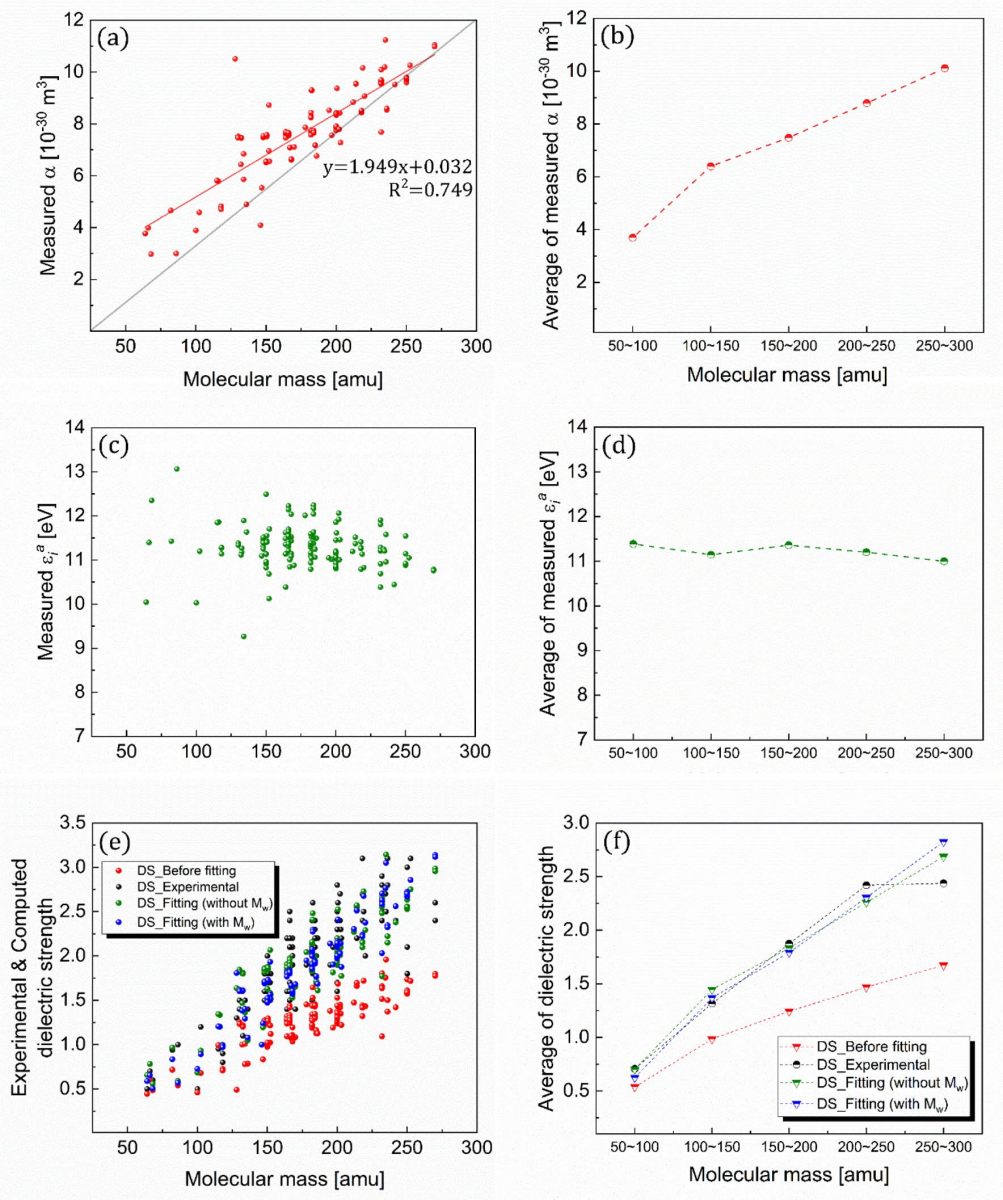
Correlations with physical property. (**a**, **b**) Polarizabilities, (**c**, **d**) ionization energies, and (**e**, **f**) dielectric strengths of the organic compounds correlated with their molecular weights.

The above-discussed intrinsic properties, such as polarizability, ionization energy, and dielectric strength, can be further correlated with the structural properties (Figs. 10 and 11). As evident from the figures, unlike the insensitivity of ionization energy to the structural properties, the polarizability increases linearly along the backbone length (the number of carbon atoms) and molecular weight. Additionally, the distinctive features of the two core variables defining dielectric strength lead to the linear correlations of the dielectric strength with the backbone length and molecular weight. These linear correlations are qualitatively applicable to all the experimental and predicted values. This suggests that the dielectric strength of an organic compound relative to the SF_6_ would rely on the difference in the polarizability between the organic compound and SF_6_, emphasizing the critical role of polarizability in determining the order of the dielectric strength.

## Conclusions

A series of well-designed computational approaches were employed in this study to design an optimal protocol that can accurately predict the dielectric strengths for a large set of organic compounds. The two fundamental variables, viz*.* polarizability and ionization energy, were computed separately for distinct sets of organic compounds using the DFT modeling approach with four distinct functional types. Since all the DFT levels of theory reliably computed values for the organic compounds showing excellent agreements with their experimental values, the B3LYP-based computational protocol was safely chosen for further investigations of the prediction of dielectric strength.

Further investigation into developing a desired protocol for predicting the dielectric strength focused on the applicability of three distinct equations that correlate dielectric strength with polarizability and ionization energy. The analysis yields three primary conclusions. First, the referenced equation developed in a previous study underrated the dielectric strengths of 137 organic compounds in our large dataset with a relative error of 30–40%. Second, four distinct equations (parameterized Eq. 1) were developed by parameterizing the coefficients of the referenced equation to reproduce the experimental dielectric strengths of the organic compounds across a range of fitting datasets. The prediction accuracy increases with the increase in the size of the fitting dataset, indicating that a fitting dataset of 137 organic compounds would be highly appropriate. Finally, the incorporation of a novel variable, viz*.* molecular weight, into the parameterized Eq. (1) revealed that a much smaller fitting dataset (30–60 organic compounds) would be sufficient for the development of the desired protocol capable of reliably predicting the dielectric strengths of the 137 organic compounds, as well as a larger dataset. All these findings highlight efforts on identifying the desired solution capable of accurately predicting the dielectric strength of an unknown organic compound.

The afore-mentioned protocols, namely (i) the reparameterization of the referenced equation and (ii) a new equation with another core variable, draw distinctive conclusions in the applications and limitations. (i) *The reparameterization of the referenced equation:* The reparameterization process based on the fitting dataset of 137 organic compounds allows us to accurately predict the dielectric strengths of the identical dataset. However, this does not guarantee the prediction ability of the protocol beyond the dataset. (ii) *A new equation with another core variable:* The parameterization process of the new equation even with smaller fitting datasets (e.g., 60 organic compounds) enables the equation to accurately predict the dielectric strengths of 137 organic compounds in the full dataset. This indicates that the newly developed equation would have an ability of the accurate prediction for unknown perfluorocarbon compounds beyond the dataset. Notably, despite such a great potential, the limitation of this protocol is an uncertainty on the reliable prediction of dielectric strengths of non-perfluorocarbon compounds.

## Supplementary Information

Supplementary Information.

## References

[1] Rabie M, Franck CM (2018). Assessment of eco-friendly gases for electrical insulation to replace the most potent industrial greenhouse gas SF6. Environ. Sci. Technol..

[2] Powell BM, Dove MT, Pawley GS, Bartell LS (1987). Orientational ordering and the low temperature structure of SF6. Mol. Phys..

[3] Maiss M, Brenninkmeijer CAM (1998). Atmospheric SF6: Trends, sources, and prospects. Environ. Sci. Technol..

[4] Qiu XQ, Chalmers ID, Coventry P (1999). A study of alternative insulating gases to SF6. J. Phys. D: Appl. Phys..

[5] Hope CW (2006). The marginal impacts of CO2, CH4 and SF6 emissions. Clim. Policy.

[6] Charlton E, Cooper F (1937). Dielectric strength of insulating fluids I. Gases and gas-vapour mixtures. Gen. Electr. Rev..

[7] Xiao S, Cressault Y, Zhang X, Teulet P (2016). The influence of Cu, Al, or Fe on the insulating capacity of CF3I. Phys.

[8] Zhang X, Xiao S, Han Y, Cressault Y (2016). Experimental studies on power frequency breakdown voltage of CF3I/N2 mixed gas under different electric fields. Appl. Phys. Lett..

[9] Pagliaro JL, Linteris GT (2017). Hydrocarbon flame inhibition by C6F12O (Novec 1230): Unstretched burning velocity measurements and predictions. Fire Saf. J..

[10] Linteris GT (2013). Unwanted combustion enhancement by C6F12O fire suppressant. Proc. Combust. Inst..

[11] Cooper, F. S. Gas-insulated electric device. (1937).

[12] Wang Y, Huang D, Liu J, Zhang Y, Zeng L (2019). Alternative environmentally friendly insulating gases for SF6. Processes.

[13] Zhang B (2020). Evaluating the dielectric strength of promising SF6 alternatives by DFT calculations and DC breakdown tests. IEEE Trans. Dielectr. Electr. Insul..

[14] Tirado-Rives J, Jorgensen WL (2008). Performance of B3LYP density functional methods for a large set of organic molecules. J. Chem. Theor. Comput..

[15] Brémond E, Adamo C (2011). Seeking for parameter-free double-hybrid functionals: The PBE0-DH model. J. Chem. Phys..

[16] Peverati R, Truhlar DG (2012). M11-L: A local density functional that provides improved accuracy for electronic structure calculations in chemistry and physics. J. Phys. Chem. Lett..

[17] Yan Z, Truhlar DG (2008). The M06 suite of density functionals for main group thermochemistry, thermochemical kinetics, noncovalent interactions, excited states, and transition elements: Two new functionals and systematic testing of four M06-class functionals and 12 other functionals. Theor. Chem. Acc..

[18] Koseki S, Schmidt MW, Gordon MS (1992). MCSCF/6-31G(d, p) calculations of one-electron spin-orbit-coupling constants in diatomic-molecules. J. Phys. Chem..

[19] Gussoni M, Rui M, Zerbi G (1998). Electronic and relaxation contribution to linear molecular polarizability. An analysis of the experimental values. J. Mol. Struct..

[20] Bartmess JE, Georgiadis RM (1990). Additivity methods in molecular polarizability. J. Am. Chem. Soc..

[21] Thakkar AJ, Wu T (2015). How well do static electronic dipole polarizabilities from gas-phase experiments compare with density functional and MP2 computations?. J. Chem. Phys..

