# Supplementary material for: Development of computational design for reliable prediction of dielectric strengths of perfluorocarbon compounds
Source: Sci Rep. 2022 Apr 29;12:7027. doi: 10.1038/s41598-022-10946-x (PMC9055060; doi:10.1038/s41598-022-10946-x)
Supplement: Supplementary file 1 — Supplementary Information. [file 41598_2022_10946_MOESM1_ESM.pdf]

Supplementary Information

Development of Computational Design for Reliable Prediction of Dielectric Strengths of Perfluorocarbon Compounds

*Joonho Jang,[1] Ku Hyun Jung[2] and Ki Chul Kim[1,2]**

J. Jang and Prof. K. C. Kim
Division of Chemical Engineering, Konkuk University, Seoul 05029, The Republic of Korea
E-mail: kich2018@konkuk.ac.kr (K. C. Kim)

K. H. Jung and Prof. K. C. Kim
Computational Materials Design Laboratory, Department of Chemical Engineering, Konkuk University, Seoul 05029, The Republic of Korea
E-mail: kich2018@konkuk.ac.kr (K. C. Kim)

Table of Contents

Figures S1 – S9: Validation of computational protocol for polarizability
Figures S10 - S17: Validation of computational protocol for ionization energy
Figures S18 – S40: Validation of computational protocol for dielectric strength

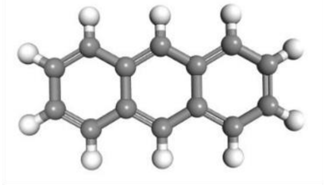

Anthracene

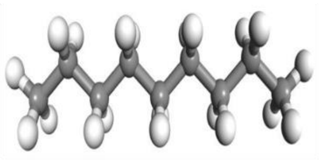

Nonane

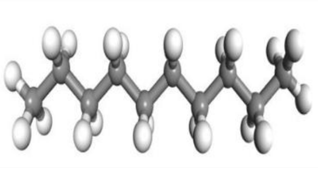

Decane

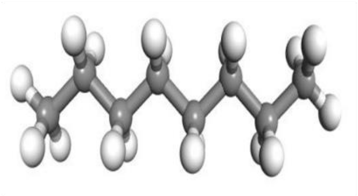

Octane

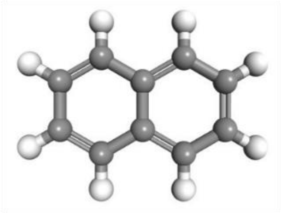

Naphthalene

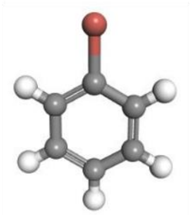

Bromobenzene

Figure S1. 1st set of organic compounds for validation of computational protocol. Chemical structures of 1st set of organic compounds introduced for the validation process of the DFT-based prediction protocol of the polarizability in Figure 2.

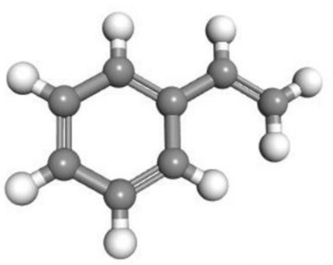

Styrene

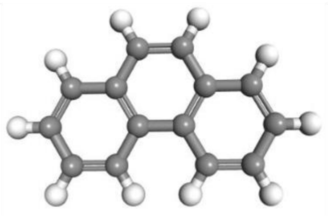

Phenanthrene

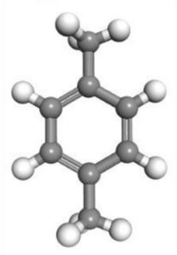

Paraxylene

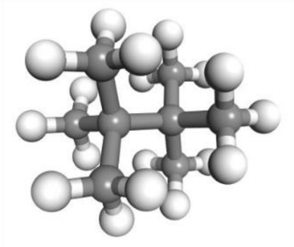

Tetramethylbutane

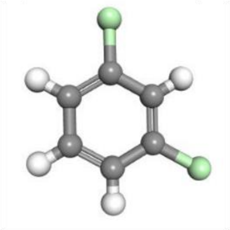

1,3-dichlorobenzene

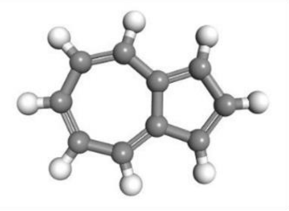

Azulene

Figure S2. 2nd set of organic compounds for validation of computational protocol. Chemical structures of 2nd set of organic compounds introduced for the validation process of the DFT-based prediction protocol of the polarizability in Figure 2.

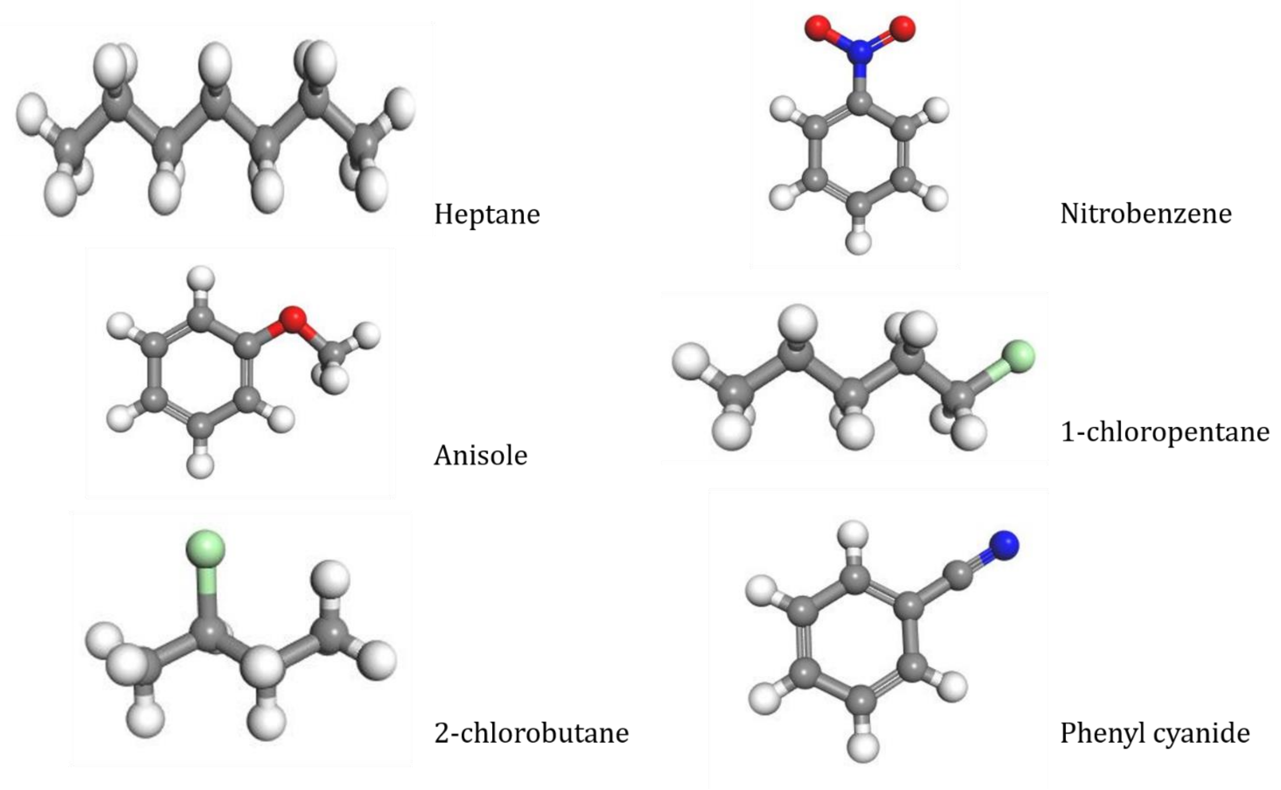

Figure S3. 3rd set of organic compounds for validation of computational protocol. Chemical structures of 3rd set of organic compounds introduced for the validation process of the DFT-based prediction protocol of the polarizability in Figure 2.

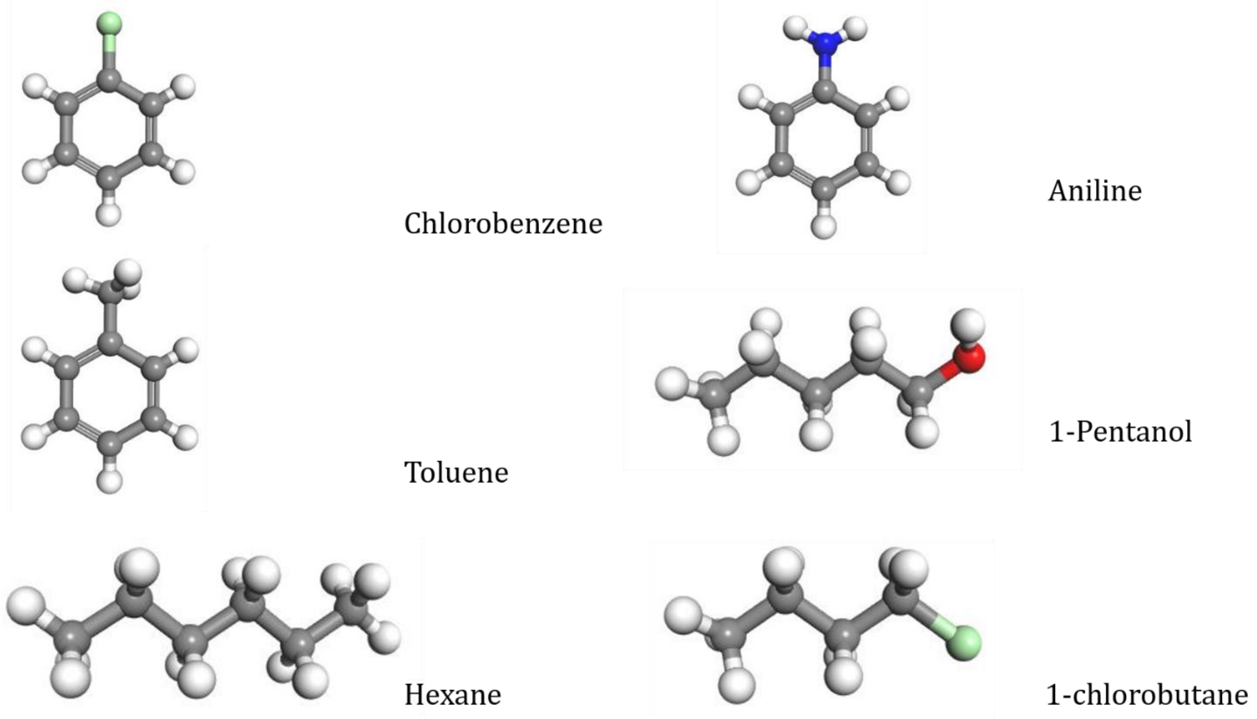

Figure S4. 4th set of organic compounds for validation of computational protocol. Chemical structures of 4th set of organic compounds introduced for the validation process of the DFT-based prediction protocol of the polarizability in Figure 2.

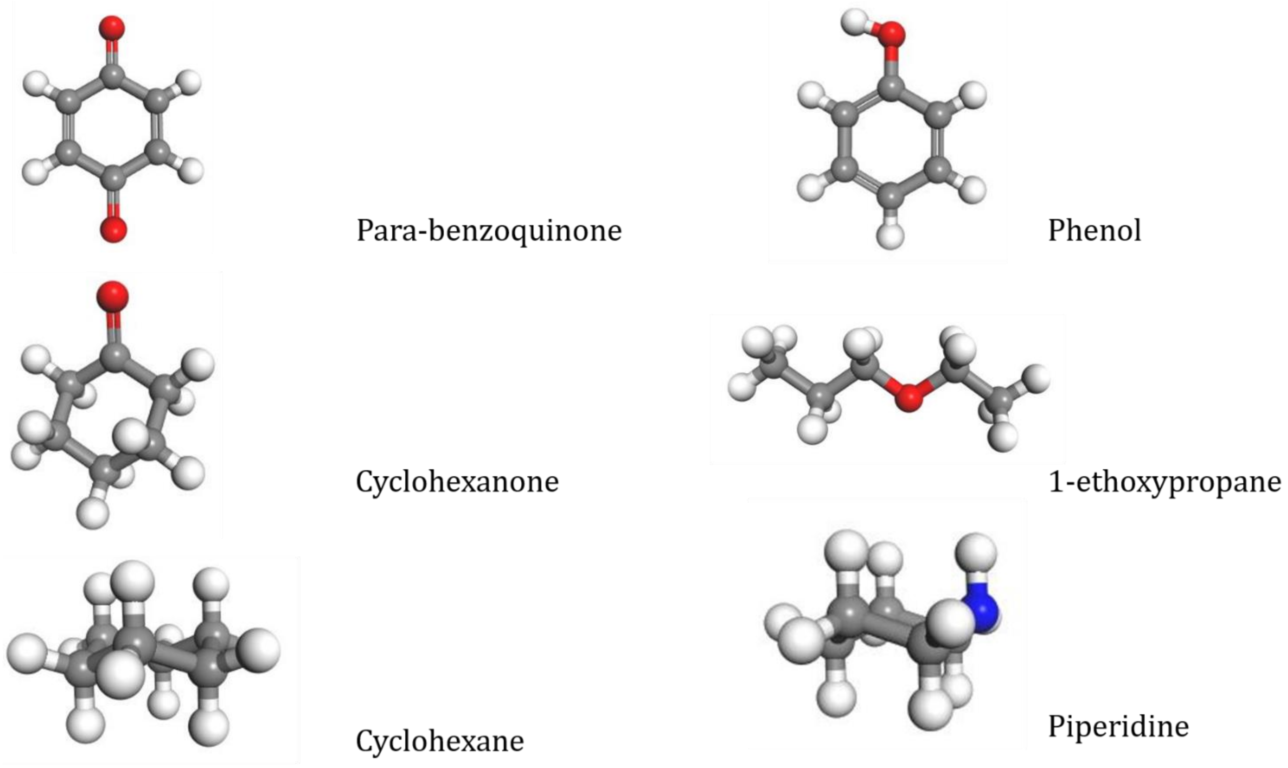

Figure S5. 5th set of organic compounds for validation of computational protocol. Chemical structures of 5th set of organic compounds introduced for the validation process of the DFT-based prediction protocol of the polarizability in Figure 2.

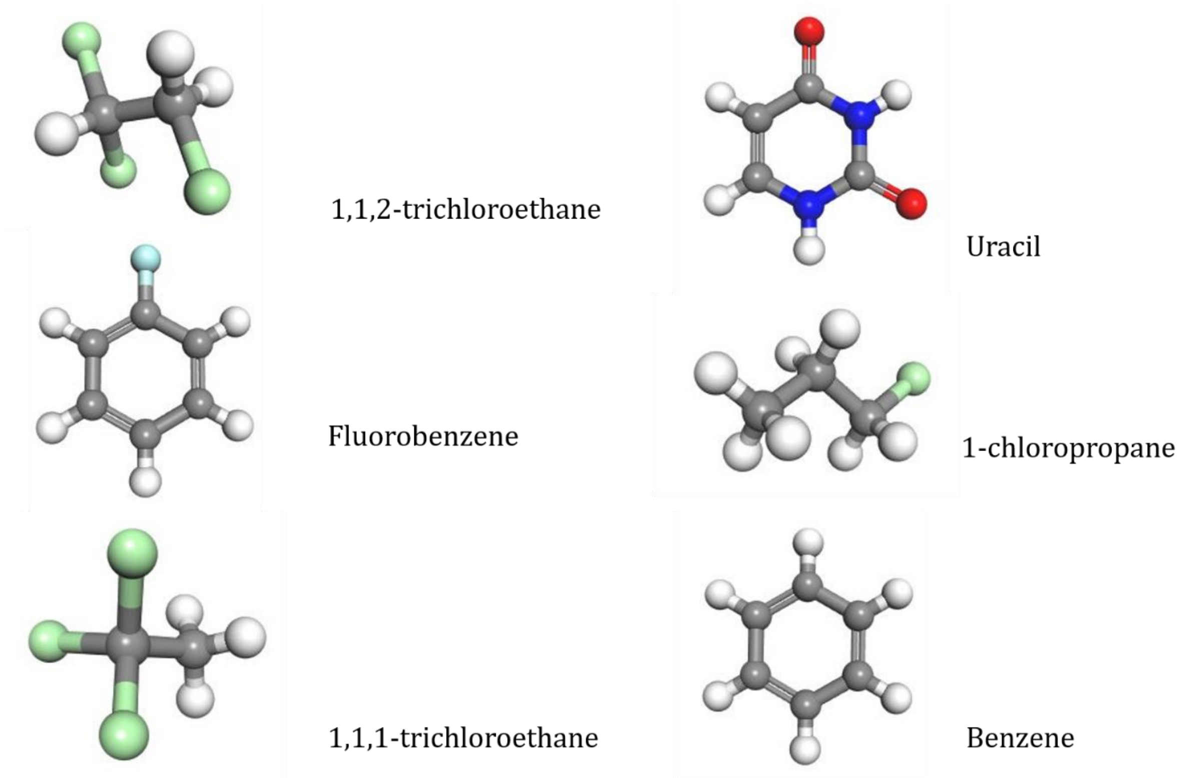

Figure S6. 6th set of organic compounds for validation of computational protocol. Chemical structures of 6th set of organic compounds introduced for the validation process of the DFT-based prediction protocol of the polarizability in Figure 2.

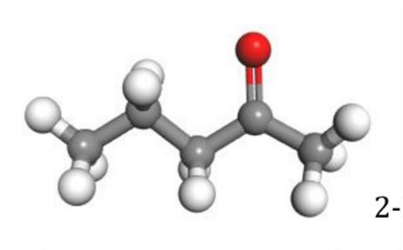

2-Pentanone

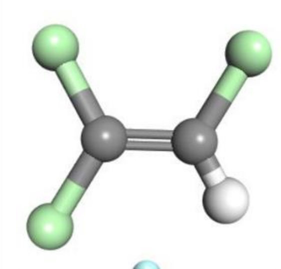

Trichloroethylene

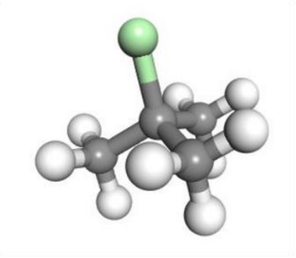

2-chloro-2-methylpropane

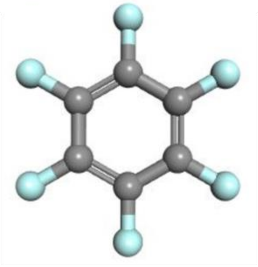

Hexafluorobenzene

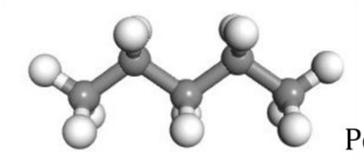

Pentane

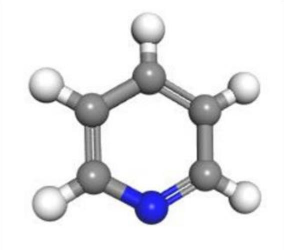

Pyridine

Figure S7. 7th set of organic compounds for validation of computational protocol. Chemical structures of 7th set of organic compounds introduced for the validation process of the DFT-based prediction protocol of the polarizability in Figure 2.

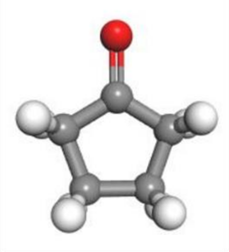

Cyclopentanone

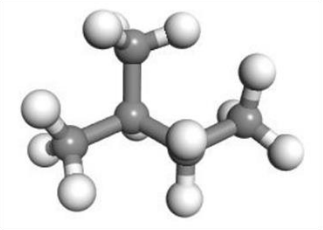

2-methylbutane

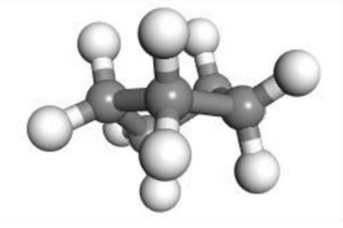

Cyclopentane

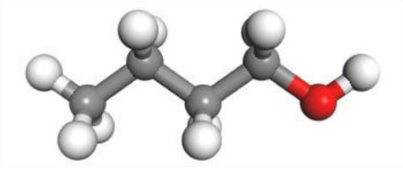

1-Butanol

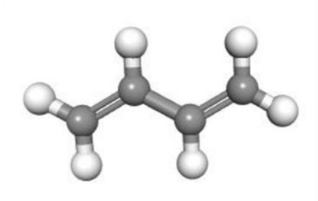

1,3-butadiene

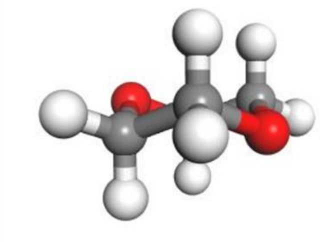

1,4-dioxane

Figure S8. 8th set of organic compounds for validation of computational protocol. Chemical structures of 8th set of organic compounds introduced for the validation process of the DFT-based prediction protocol of the polarizability in Figure 2.

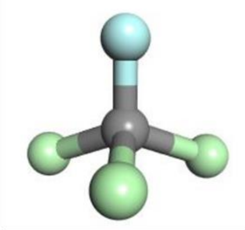

Trichloromonofluoromethane

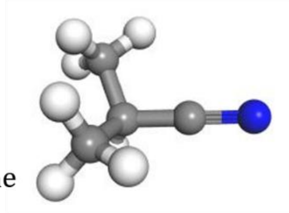

2-methylpropanenitrile

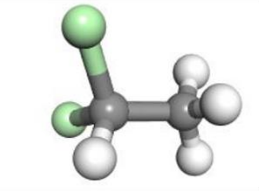

1,1-dichloroethane

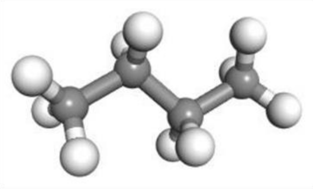

Butane

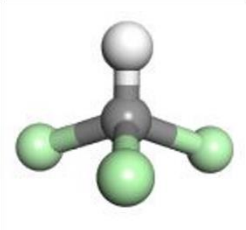

Chloroform

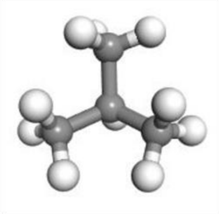

Isobutane

Figure S9. 9th set of organic compounds for validation of computational protocol. Chemical structures of 9th set of organic compounds introduced for the validation process of the DFT-based prediction protocol of the polarizability in Figure 2.

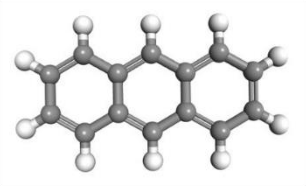

Anthracene

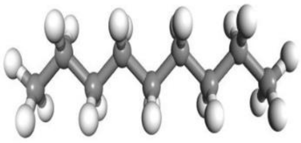

Nonane

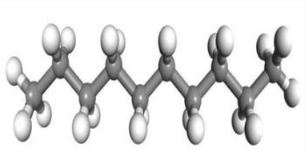

Decane

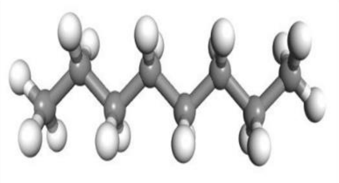

Octane

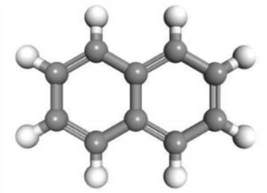

Naphthalene

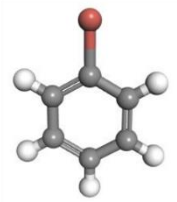

Bromobenzene

Figure S10. 1st set of organic compounds for validation of computational protocol. Chemical structures of 1st set of organic compounds introduced for the validation process of the DFT-based prediction protocol of the ionization energy in Figure 3.

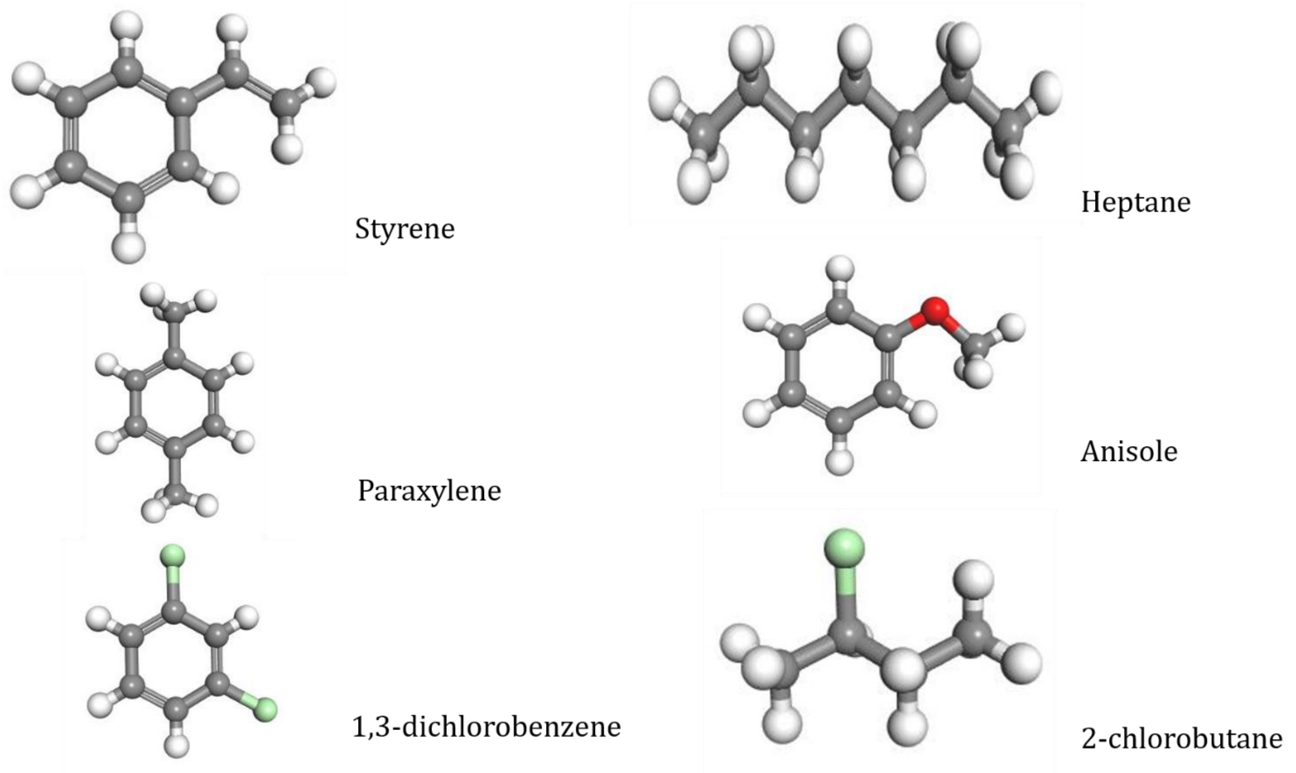

Figure S11. 2nd set of organic compounds for validation of computational protocol. Chemical structures of 2nd set of organic compounds introduced for the validation process of the DFT-based prediction protocol of the ionization energy in Figure 3.

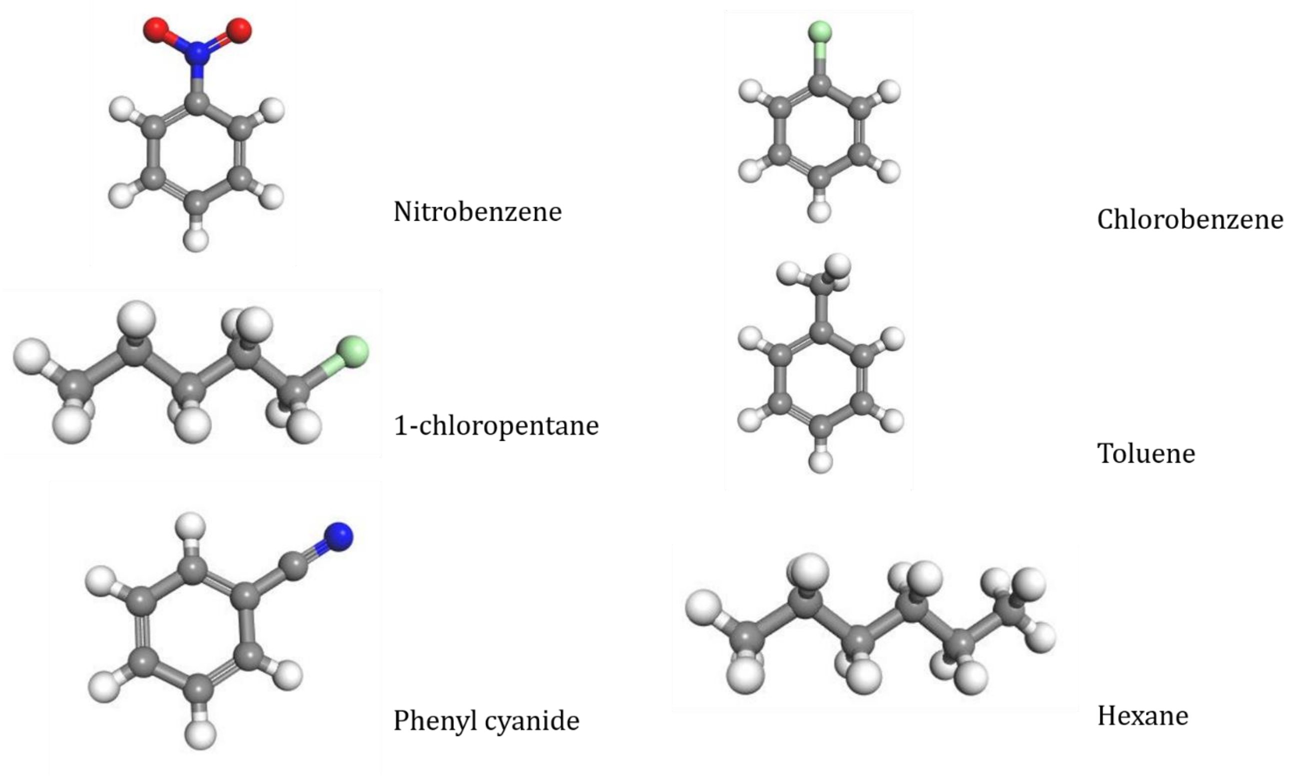

Figure S12. 3rd set of organic compounds for validation of computational protocol. Chemical structures of 3rd set of organic compounds introduced for the validation process of the DFT-based prediction protocol of the ionization energy in Figure 3.

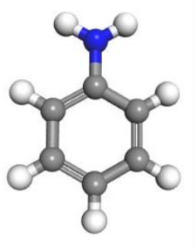

Aniline

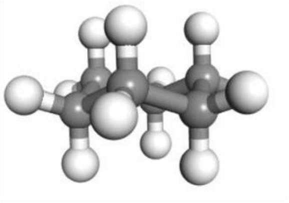

Cyclohexane

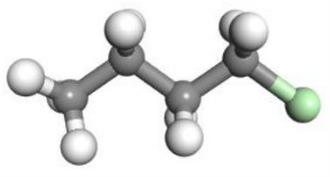

1-chlorobutane

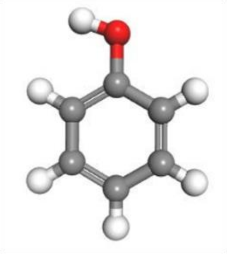

Phenol

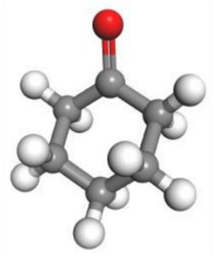

Cyclohexanone

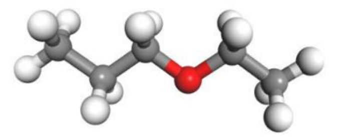

1-ethoxypropane

Figure S13. 4th set of organic compounds for validation of computational protocol. Chemical structures of 4th set of organic compounds introduced for the validation process of the DFT-based prediction protocol of the ionization energy in Figure 3.

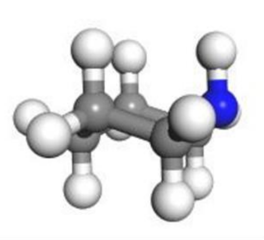

Piperidine

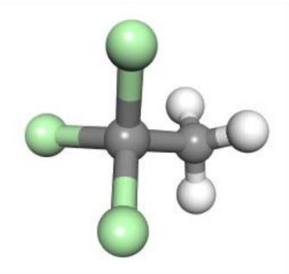

1,1,1-trichloroethane

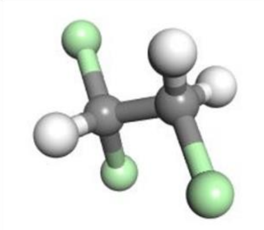

1,1,2-trichloroethane

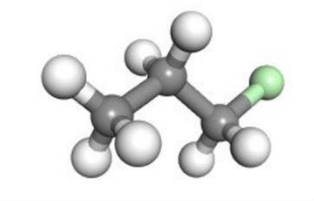

1-chloropropane

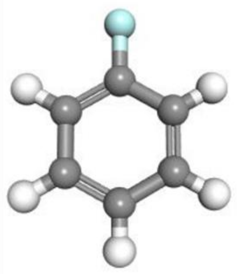

Fluorobenzene

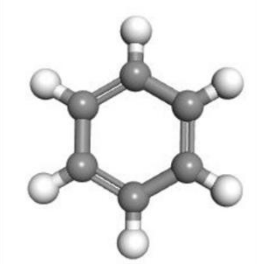

Benzene

Figure S14. 5th set of organic compounds for validation of computational protocol. Chemical structures of 5th set of organic compounds introduced for the validation process of the DFT-based prediction protocol of the ionization energy in Figure 3.

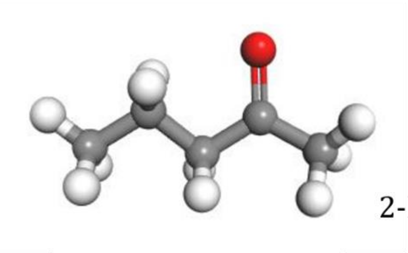

2-Pentanone

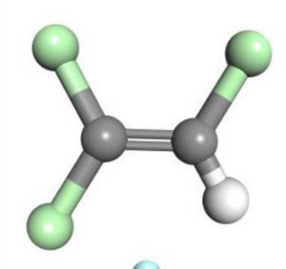

Trichloroethylene

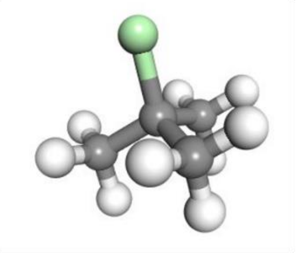

2-chloro-2-methylpropane

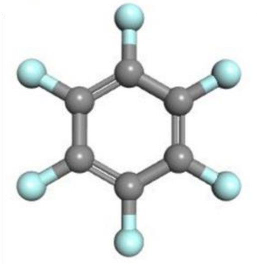

Hexafluorobenzene

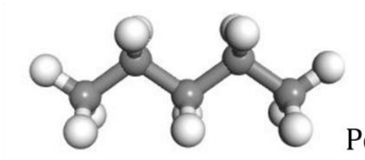

Pentane

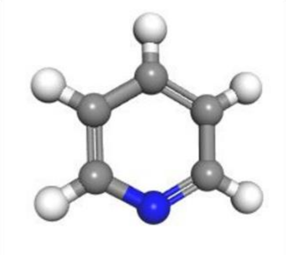

Pyridine

Figure S15. 6th set of organic compounds for validation of computational protocol. Chemical structures of 6th set of organic compounds introduced for the validation process of the DFT-based prediction protocol of the ionization energy in Figure 3.

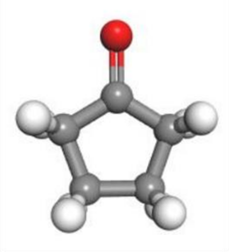

Cyclopentanone

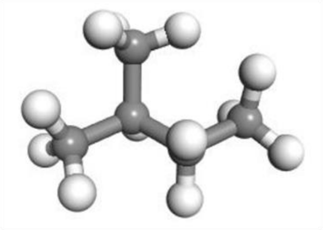

2-methylbutane

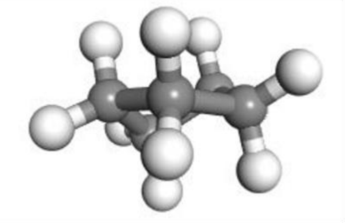

Cyclopentane

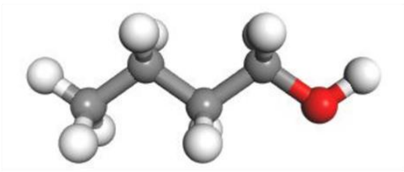

1-Butanol

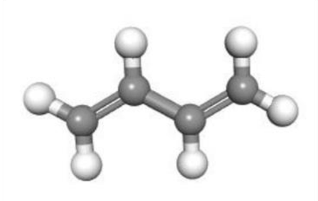

1,3-butadiene

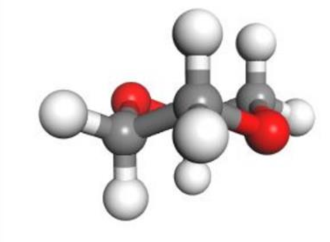

1,4-dioxane

Figure S16. 7th set of organic compounds for validation of computational protocol. Chemical structures of 7th set of organic compounds introduced for the validation process of the DFT-based prediction protocol of the ionization energy in Figure 3.

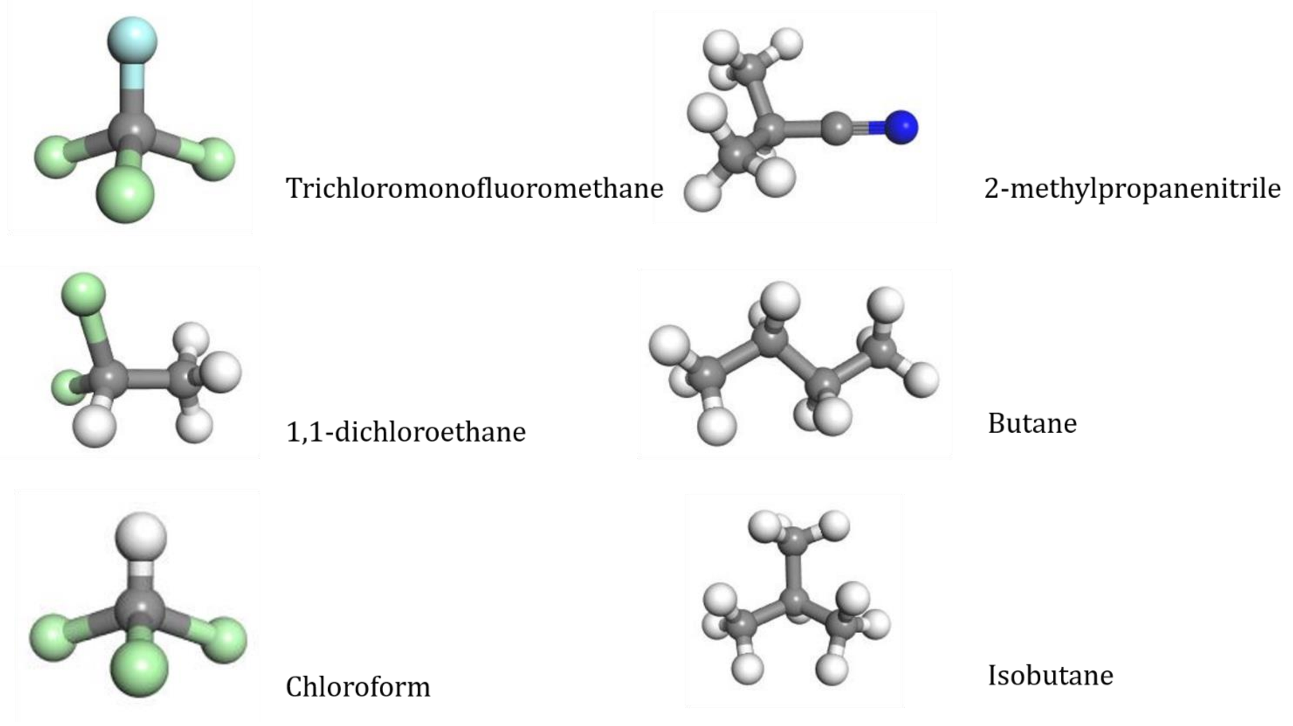

Figure S17. 8th set of organic compounds for validation of computational protocol. Chemical structures of 8th set of organic compounds introduced for the validation process of the DFT-based prediction protocol of the ionization energy in Figure 3.

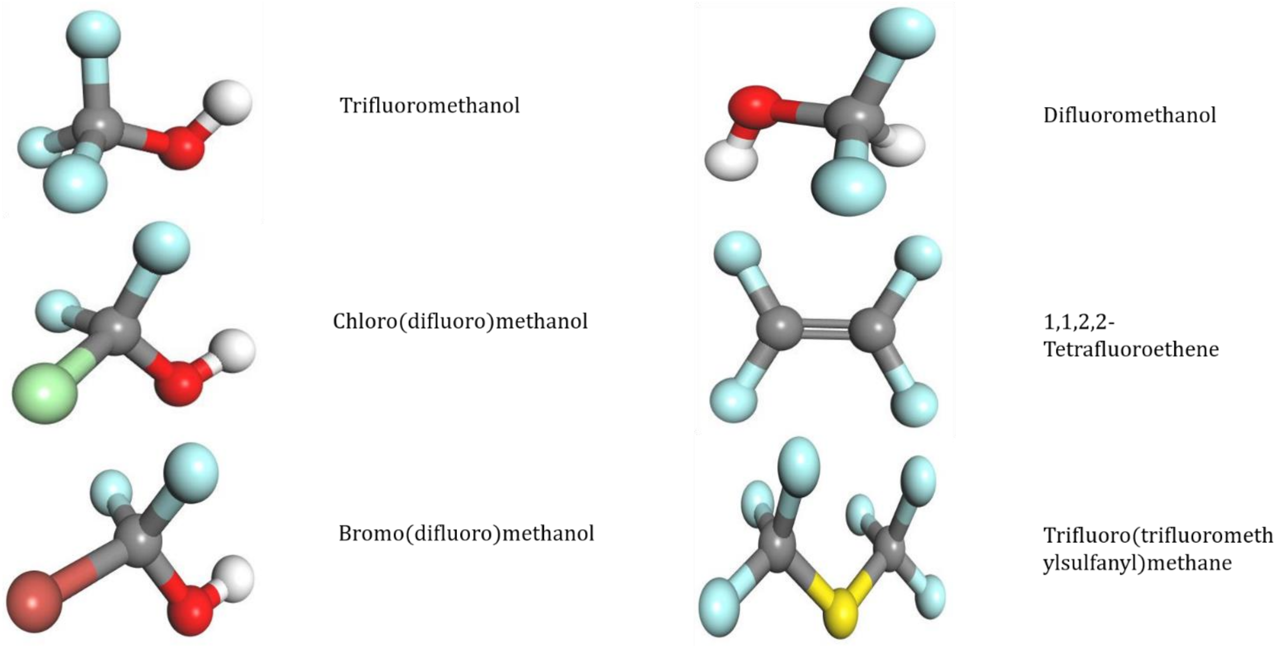

Figure S18. 1st set of organic compounds for validation of computational protocol. Chemical structures of 1st set of organic compounds introduced for the validation process of the DFT-based prediction protocol of the dielectric strength in Figure 5.

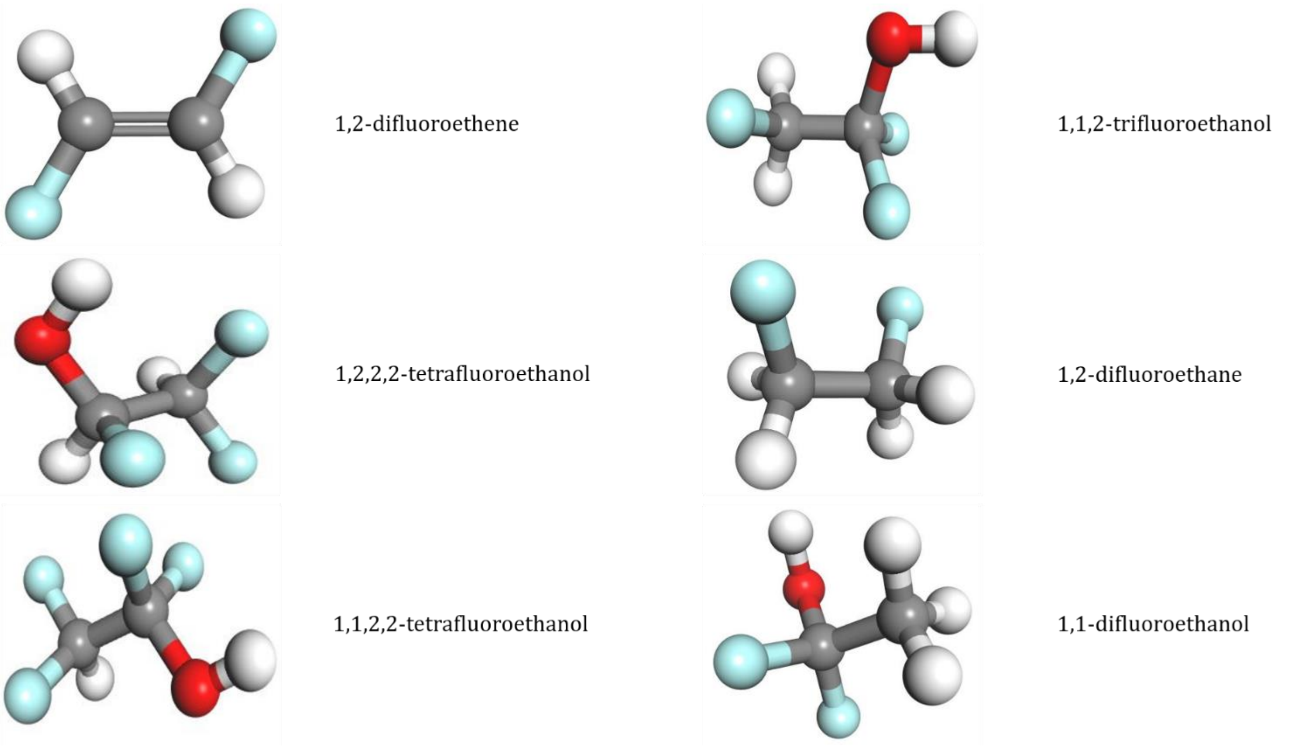

Figure S19. 2nd set of organic compounds for validation of computational protocol. Chemical structures of 2nd set of organic compounds introduced for the validation process of the DFT-based prediction protocol of the dielectric strength in Figure 5.

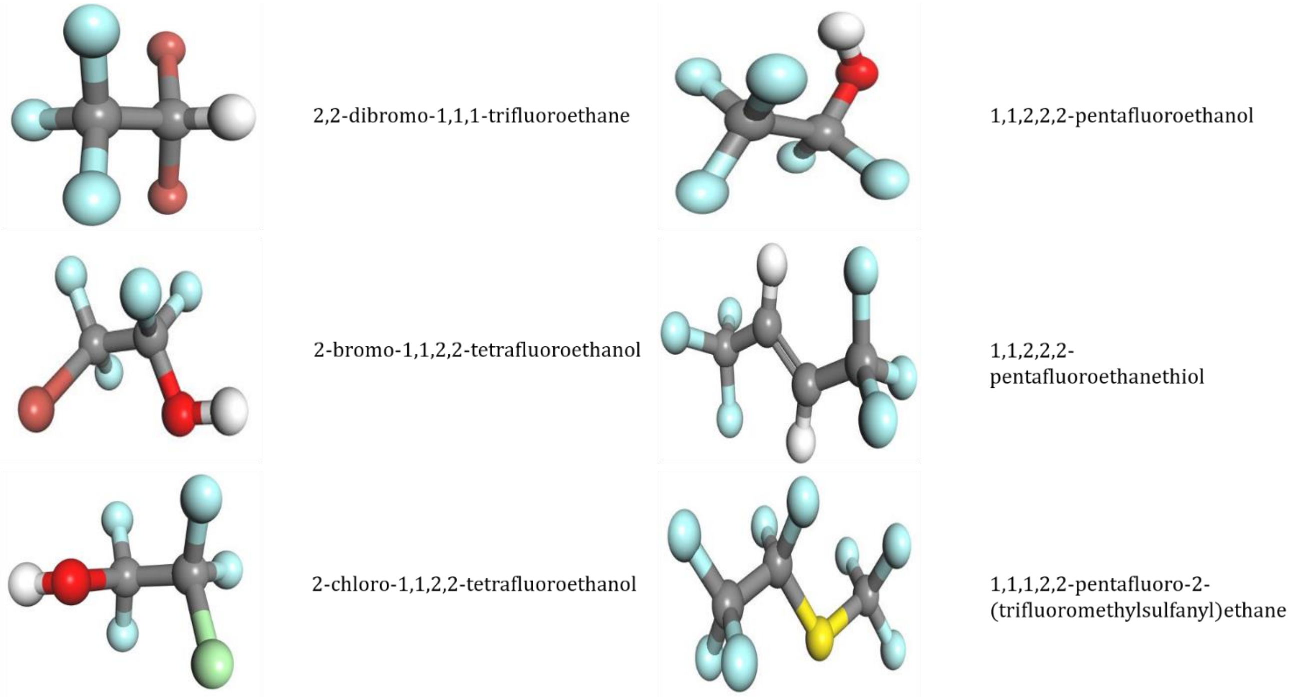

Figure S20. 3rd set of organic compounds for validation of computational protocol. Chemical structures of 3rd set of organic compounds introduced for the validation process of the DFT-based prediction protocol of the dielectric strength in Figure 5.

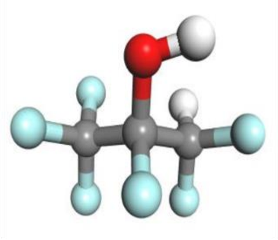

(2S)-1,1,1,2,3,3-hexafluoropropan-2-ol

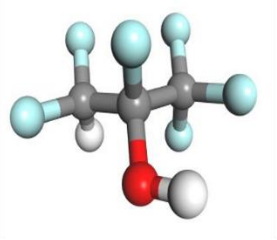

1,1,1,2,3,3-hexafluoropropan-2-ol

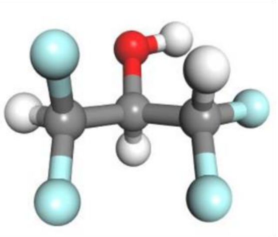

1,1,1,3,3,3-hexafluoropropan-2-ol

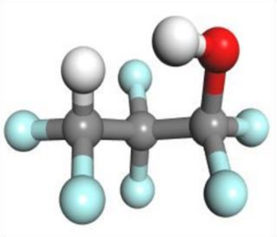

1,1,2,2,3,3-hexafluoropropan-1-ol

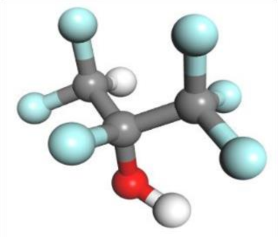

(2R)-1,1,1,2,3,3-hexafluoropropan-2-ol

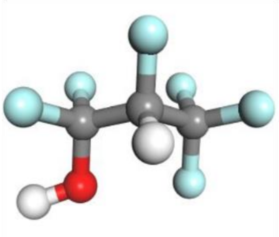

1,1,2,3,3,3-hexafluoropropan-1-ol

Figure S21. 4th set of organic compounds for validation of computational protocol. Chemical structures of 4th set of organic compounds introduced for the validation process of the DFT-based prediction protocol of the dielectric strength in Figure 5.

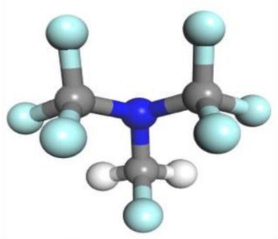

1,1,1-trifluoro-N-(fluoromethyl)-N-(trifluoromethyl)methanamine

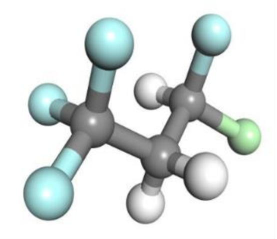

3-chloro-1,1,1,3-tetrafluoropropane

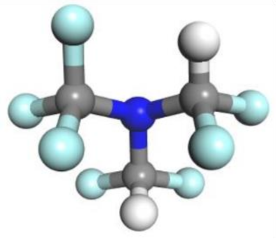

N,N-bis(difluoromethyl)-1,1,1-trifluoromethanamine

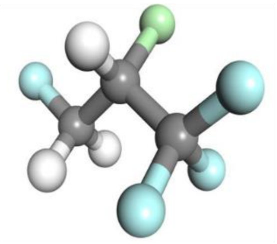

2-chloro-1,1,1,3-tetrafluoropropane

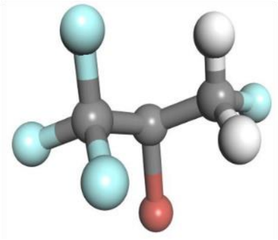

2-bromo-1,1,1,3-tetrafluoropropane

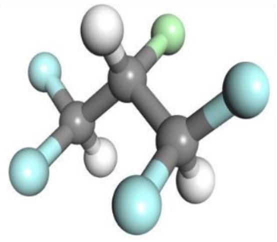

2-chloro-1,1,3,3-tetrafluoropropane

Figure S22. 5th set of organic compounds for validation of computational protocol. Chemical structures of 5th set of organic compounds introduced for the validation process of the DFT-based prediction protocol of the dielectric strength in Figure 5.

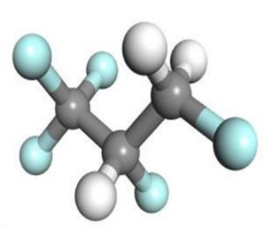

(2R)-1,1,1,2,3-pentafluoropropane

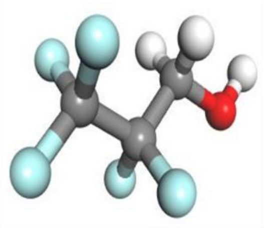

2,2,3,3,3-pentafluoropropan-1-ol

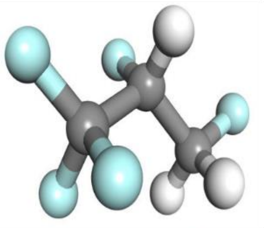

1,1,1,2,3-pentafluoropropane

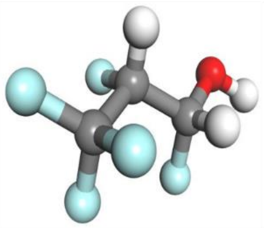

1,2,3,3,3-pentafluoropropan-1-ol

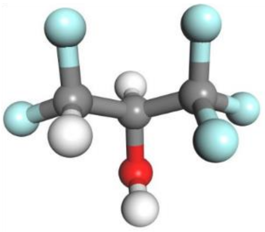

1,1,1,3,3-pentafluoropropan-2-ol

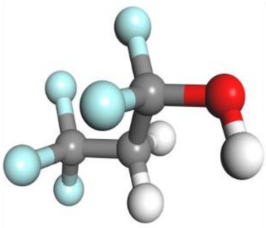

1,1,3,3,3-pentafluoropropan-1-ol

Figure S23. 6th set of organic compounds for validation of computational protocol. Chemical structures of 6th set of organic compounds introduced for the validation process of the DFT-based prediction protocol of the dielectric strength in Figure 5.

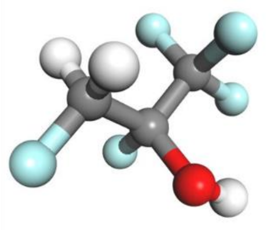

1,1,1,2,3-pentafluoropropan-2-ol

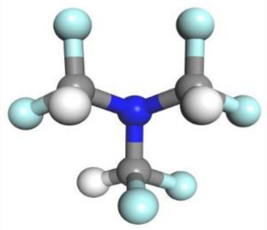

N,N-bis(difluoromethyl)-1,1-difluoromethanamine

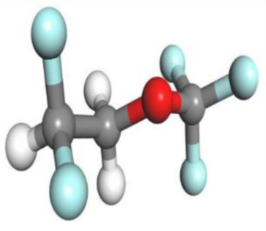

1,1-difluoro-2-(trifluoromethoxy)ethane

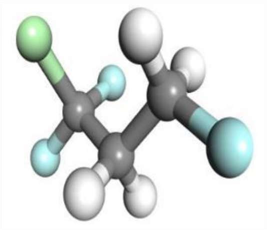

1-chloro-1,1,3-trifluoropropane

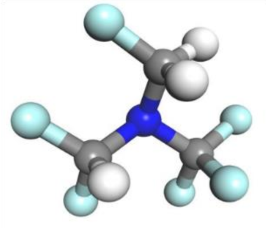

N-(difluoromethyl)-1,1,1-trifluoro-N-(fluoromethyl)methanamine

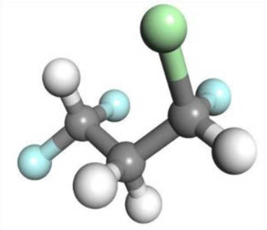

1-chloro-1,3,3-trifluoropropane

Figure S24. 7th set of organic compounds for validation of computational protocol. Chemical structures of 7th set of organic compounds introduced for the validation process of the DFT-based prediction protocol of the dielectric strength in Figure 5.

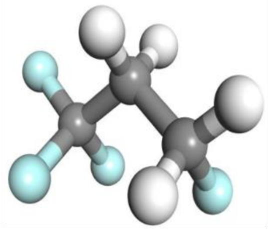

1,1,1,3-tetrafluoropropane

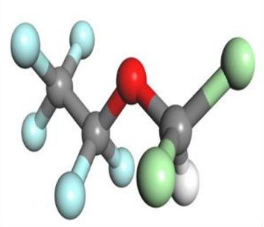

1-(dichloromethoxy)-
1,1,2,2,2-pentafluoroethane

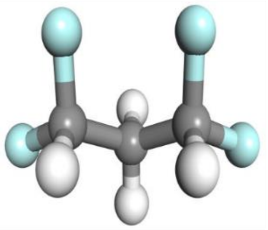

1,1,3,3-tetrafluoropropane

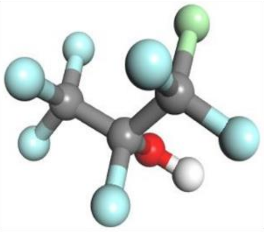

1-chloro-1,1,2,3,3,3-
hexafluoropropan-2-ol

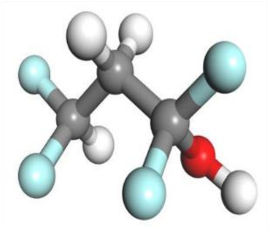

1,1,3,3-tetrafluoropropan-1-ol

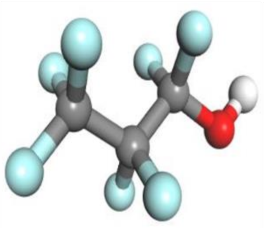

1,1,2,2,3,3,3-
heptafluoropropan-1-ol

Figure S25. 8th set of organic compounds for validation of computational protocol. Chemical structures of 8th set of organic compounds introduced for the validation process of the DFT-based prediction protocol of the dielectric strength in Figure 5.

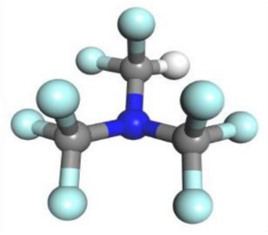

N-(difluoromethyl)-1,1,1-trifluoro-
N-(trifluoromethyl)methanamine

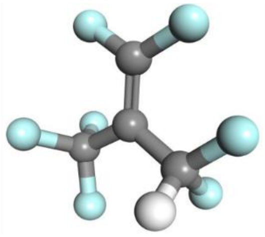

2-(difluoromethyl)-1,1,3,3,3-
pentafluoroprop-1-ene

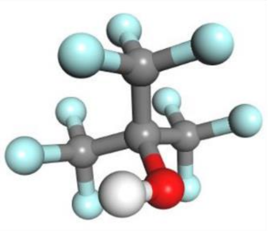

1,1,1,3,3,3-hexafluoro-2-
(trifluoromethyl)propan-2-ol

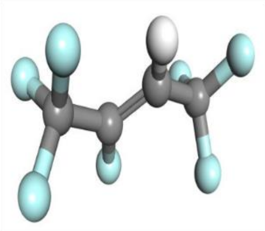

(Z)-1,1,1,2,4,4,4-
heptafluorobut-2-ene

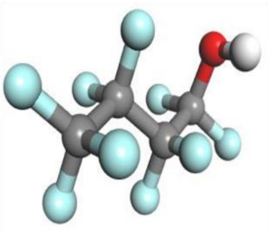

1,1,2,2,3,3,4,4,4-nonafluorobutan-1-ol

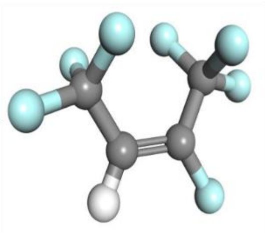

(E)-1,1,1,2,4,4,4-
heptafluorobut-2-ene

Figure S26. 9th set of organic compounds for validation of computational protocol. Chemical structures of 9th set of organic compounds introduced for the validation process of the DFT-based prediction protocol of the dielectric strength in Figure 5.

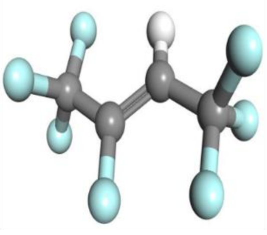

1,1,1,2,4,4,4-heptafluorobut-2-ene

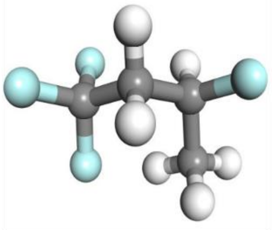

1,1,1,3-tetrafluorobutane

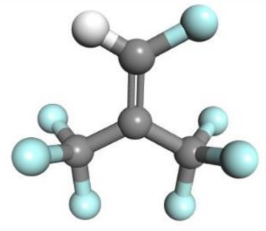

1,3,3,3-tetrafluoro-2-(trifluoromethyl)prop-1-ene

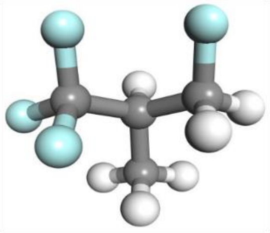

1,1,1,3-tetrafluoro-2-methylpropane

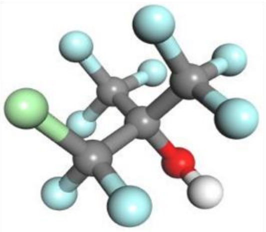

1-chloro-1,1,3,3,3-pentafluoro-2-(trifluoromethyl)propan-2-ol

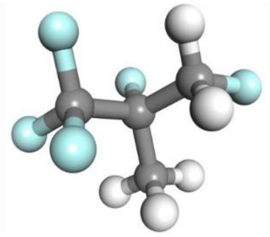

1,1,1,2,3-pentafluoro-2-methylpropane

Figure S27. 10th set of organic compounds for validation of computational protocol. Chemical structures of 10th set of organic compounds introduced for the validation process of the DFT-based prediction protocol of the dielectric strength in Figure 5.

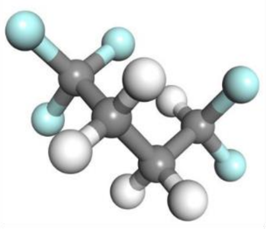

1,1,1,4,4-pentafluorobutane

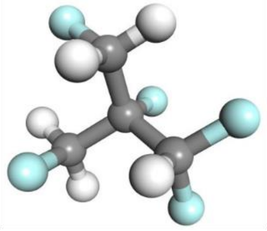

1,1,2,3-tetrafluoro-2-(fluoromethyl)propane

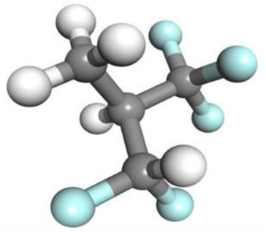

1,1,1,3,3-pentafluoro-2-methylpropane

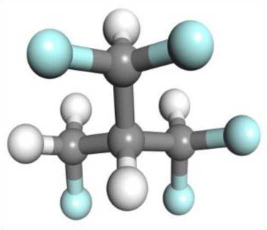

1,1,3,3-tetrafluoro-2-(fluoromethyl)propane

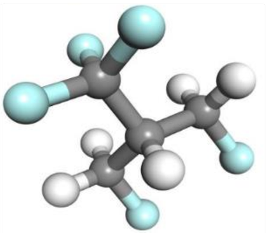

1,1,1,3-tetrafluoro-2-(fluoromethyl)propane

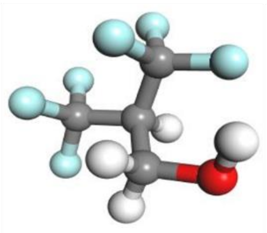

3,3,3-trifluoro-2-(trifluoromethyl)propan-1-ol

Figure S28. 11th set of organic compounds for validation of computational protocol. Chemical structures of 11th set of organic compounds introduced for the validation process of the DFT-based prediction protocol of the dielectric strength in Figure 5.

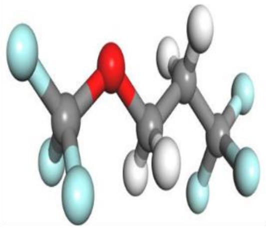

1,1,1-trifluoro-3-(trifluoromethoxy)propane

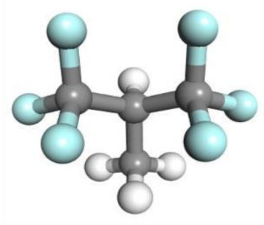

1,1,1,3,3,3-hexafluoro-2-methylpropane

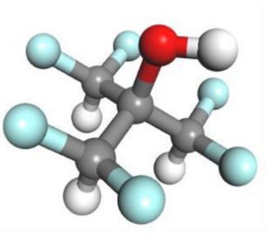

2-(difluoromethyl)-1,1,3,3-tetrafluoropropan-2-ol

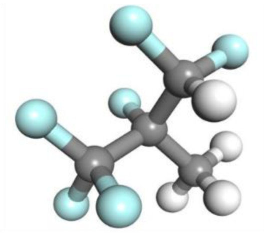

1,1,1,2,3,3-hexafluoro-2-methylpropane

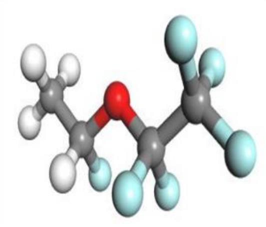

1,1,1,2,2-pentafluoro-2-(1-fluoroethoxy)ethane

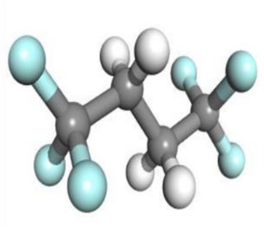

1,1,1,4,4,4-hexafluorobutane

Figure S29. 12th set of organic compounds for validation of computational protocol. Chemical structures of 12th set of organic compounds introduced for the validation process of the DFT-based prediction protocol of the dielectric strength in Figure 5.

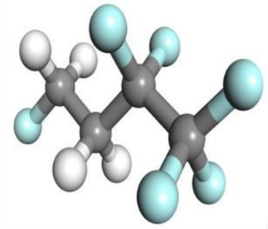

1,1,1,2,2,4-hexafluorobutane

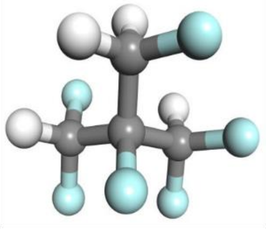

1,1,2,3,3-pentafluoro-2-(fluoromethyl)propane

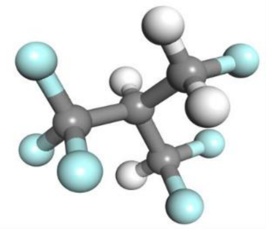

1,1,1,3,3-pentafluoro-2-(fluoromethyl)propane

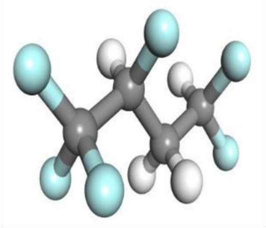

1,1,1,2,4,4-hexafluorobutane

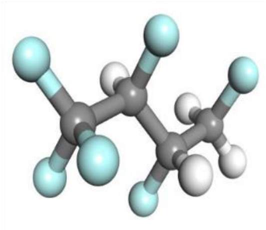

1,1,1,2,3,4-hexafluorobutane

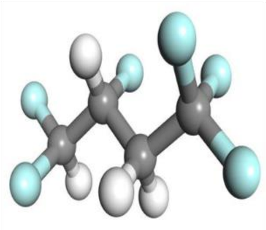

1,1,1,3,4,4-hexafluorobutane

Figure S30. 13th set of organic compounds for validation of computational protocol. Chemical structures of 13th set of organic compounds introduced for the validation process of the DFT-based prediction protocol of the dielectric strength in Figure 5.

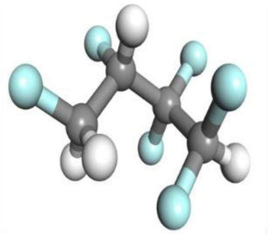

1,1,2,2,3,4-hexafluorobutane

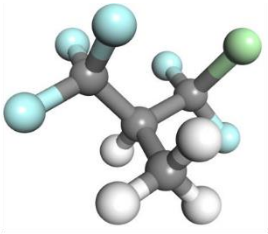

1-chloro-1,1,3,3,3-pentafluoro-2-methylpropane

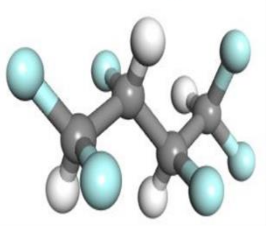

1,1,2,3,4,4-hexafluorobutane

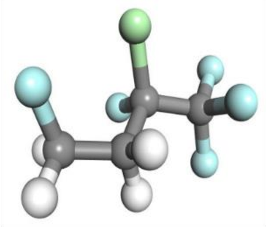

2-chloro-1,1,1,2,4-pentafluorobutane

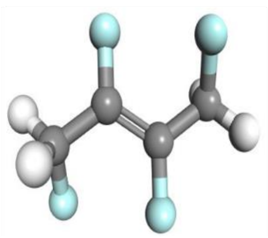

(E)-1,2,3,4-tetrafluorobut-2-ene

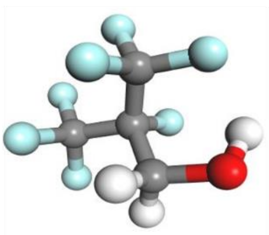

2,3,3,3-tetrafluoro-2-(trifluoromethyl)propan-1-ol

Figure S31. 14th set of organic compounds for validation of computational protocol. Chemical structures of 14th set of organic compounds introduced for the validation process of the DFT-based prediction protocol of the dielectric strength in Figure 5.

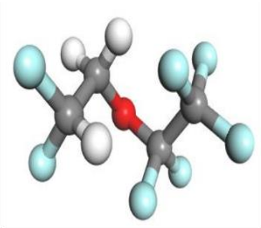

1-(2,2-difluoroethoxy)-1,1,2,2,2-pentafluoroethane

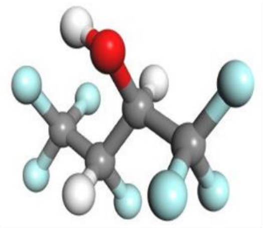

1,1,1,3,4,4,4-heptafluorobutan-2-ol

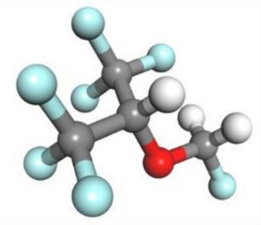

1,1,1,3,3,3-hexafluoro-2-(fluoromethoxy)propane

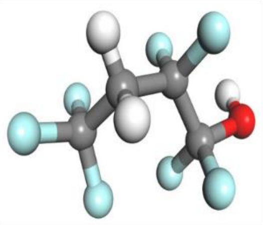

1,1,2,2,4,4,4-heptafluorobutan-1-ol

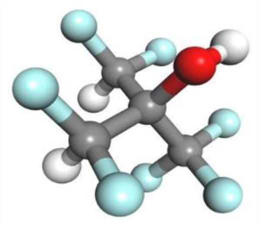

2-(difluoromethyl)-1,1,1,3,3-pentafluoropropan-2-ol

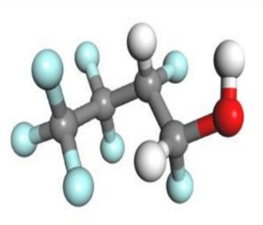

1,2,3,3,4,4,4-heptafluorobutan-1-ol

Figure S32. 15th set of organic compounds for validation of computational protocol. Chemical structures of 15th set of organic compounds introduced for the validation process of the DFT-based prediction protocol of the dielectric strength in Figure 5.

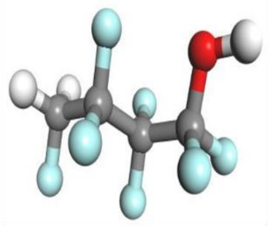

1,1,2,2,3,3,4-heptafluorobutan-1-ol

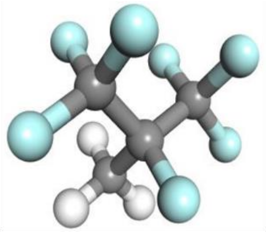

1,1,1,2,3,3,3-heptafluoro-2-methylpropane

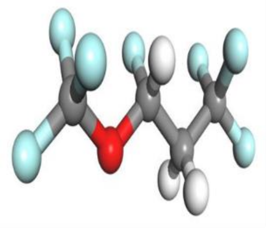

1,1,1,3-tetrafluoro-3-(trifluoromethoxy)propane

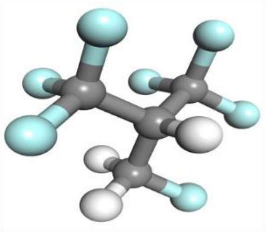

1,1,1,3,3,3-hexafluoro-2-(fluoromethyl)propane

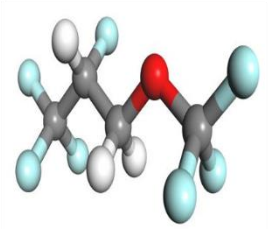

1,1,1,2-tetrafluoro-3-(trifluoromethoxy)propane

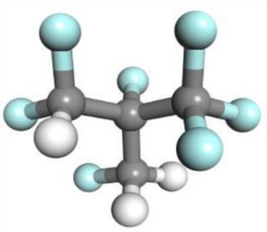

1,1,1,2,3,3-hexafluoro-2-(fluoromethyl)propane

Figure S33. 16th set of organic compounds for validation of computational protocol. Chemical structures of 16th set of organic compounds introduced for the validation process of the DFT-based prediction protocol of the dielectric strength in Figure 5.

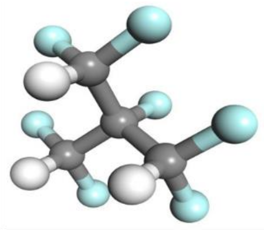

2-(difluoromethyl)-1,1,2,3,3-pentafluoropropane

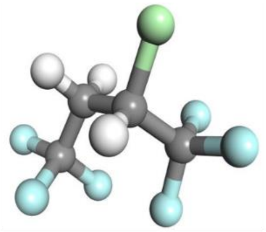

2-chloro-1,1,1,4,4,4-hexafluorobutane

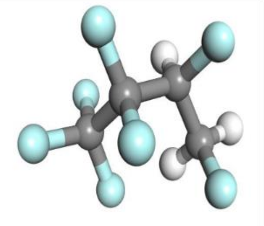

1,1,1,2,2,3,4-heptafluorobutane

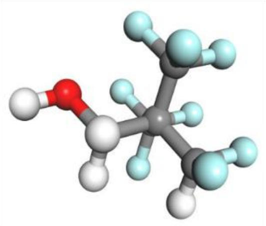

2-(difluoromethyl)-1,1,1,3,3,3-hexafluoropropan-2-ol

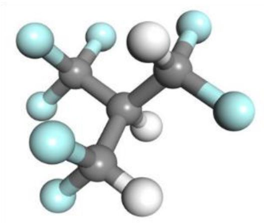

2-(difluoromethyl)-1,1,1,3,3-pentafluoropropane

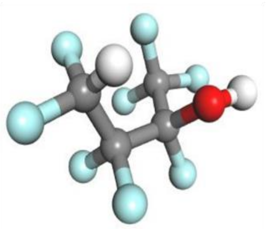

1,1,1,2,3,3,4,4-octafluorobutan-2-ol

Figure S34. 17th set of organic compounds for validation of computational protocol. Chemical structures of 17th set of organic compounds introduced for the validation process of the DFT-based prediction protocol of the dielectric strength in Figure 5.

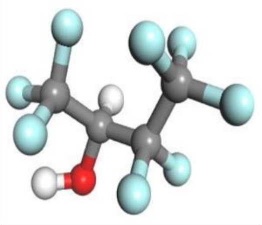

1,1,1,3,3,4,4,4-octafluorobutan-2-ol

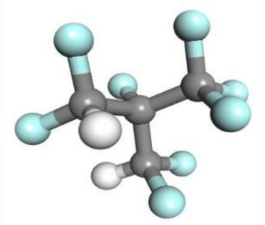

2-(difluoromethyl)-1,1,1,2,3,3-hexafluoropropane

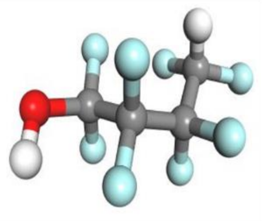

1,1,2,2,3,3,4,4-octafluorobutan-1-ol

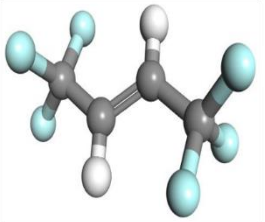

(E)-1,1,1,4,4,4-hexafluorobut-2-ene

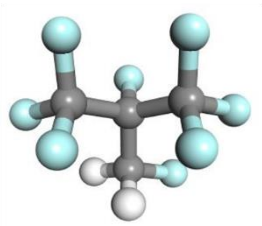

1,1,1,2,3,3,3-heptafluoro-2-(fluoromethyl)propane

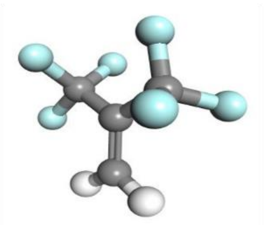

1,1,1,4,4,4-hexafluorobut-2-ene

Figure S35. 18th set of organic compounds for validation of computational protocol. Chemical structures of 18th set of organic compounds introduced for the validation process of the DFT-based prediction protocol of the dielectric strength in Figure 5.

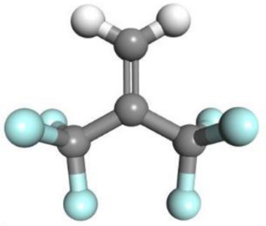

3,3,3-trifluoro-2-(trifluoromethyl)prop-1-ene

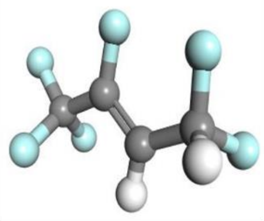

1,1,1,2,4,4-hexafluorobut-2-ene

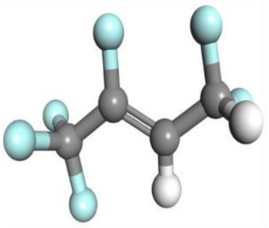

1,1,1,2,4,4-hexafluorobut-2-ene

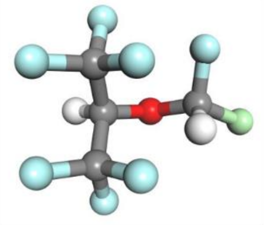

2-[chloro(fluoro)methoxy]-1,1,1,3,3,3-hexafluoropropane

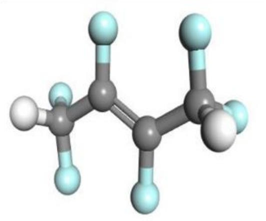

(E)-1,1,2,3,4,4-hexafluorobut-2-ene

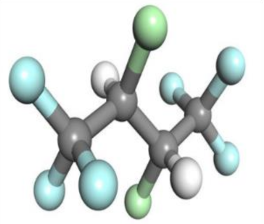

2,3-dichloro-1,1,1,4,4,4-hexafluorobutane

Figure S36. 19th set of organic compounds for validation of computational protocol. Chemical structures of 19th set of organic compounds introduced for the validation process of the DFT-based prediction protocol of the dielectric strength in Figure 5.

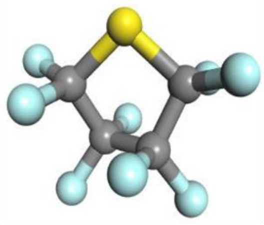

2,2,3,3,4,4,5,5-octafluorothiolane

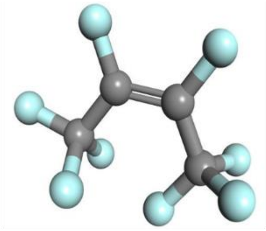

(Z)-1,1,1,2,3,4,4,4-octafluorobut-2-ene

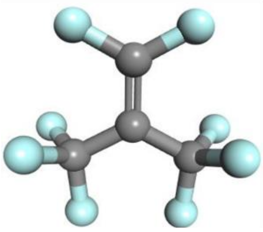

1,1,3,3,3-pentafluoro-2-(trifluoromethyl)prop-1-ene

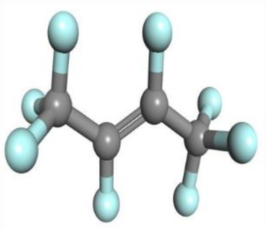

1,1,1,2,3,4,4,4-octafluorobut-2-ene

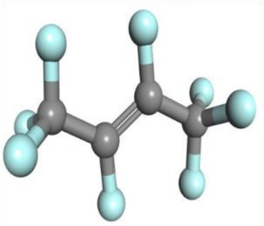

(E)-1,1,1,2,3,4,4,4-octafluorobut-2-ene

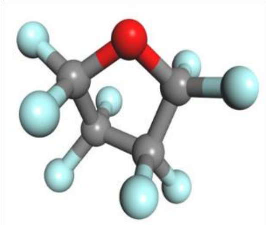

2,2,3,4,5,5-hexafluorofuran

Figure S37. 20th set of organic compounds for validation of computational protocol. Chemical structures of 20th set of organic compounds introduced for the validation process of the DFT-based prediction protocol of the dielectric strength in Figure 5.

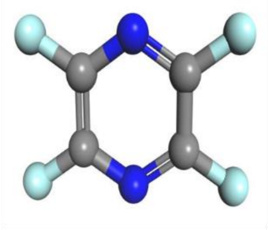

2,3,5,6-tetrafluoropyrazine

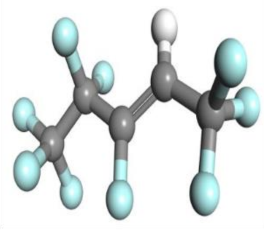

1,1,1,3,4,4,5,5,5-nonafluoropent-2-ene

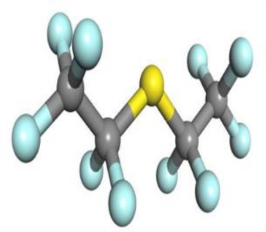

1,1,1,2,2-pentafluoro-2-(1,1,2,2,2-pentafluoroethylsulfanyl)ethane

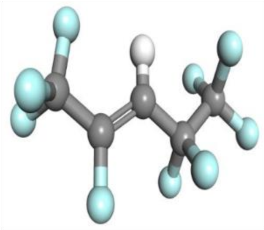

1,1,1,2,4,4,5,5,5-nonafluoropent-2-ene

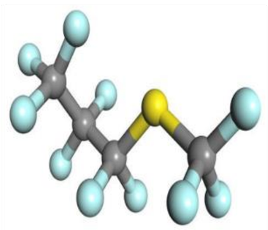

1,1,1,2,2,3,3-heptafluoro-3-(trifluoromethylsulfanyl)propane

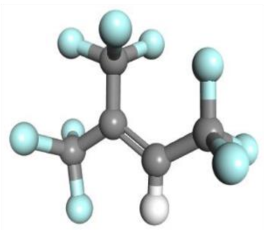

1,1,1,4,4,4-hexafluoro-2-(trifluoromethyl)but-2-ene

Figure S38. 21th set of organic compounds for validation of computational protocol. Chemical structures of 21th set of organic compounds introduced for the validation process of the DFT-based prediction protocol of the dielectric strength in Figure 5.

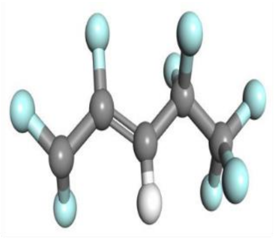

(E)-1,1,1,3,4,4,5,5,5-nonafluoropent-2-ene

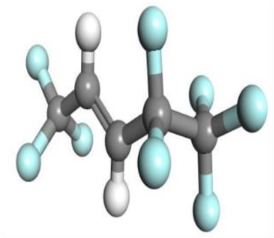

(E)-1,1,1,4,4,5,5,5-octafluoropent-2-ene

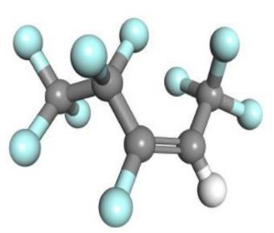

(E)-1,1,1,2,4,4,5,5,5-nonafluoropent-2-ene

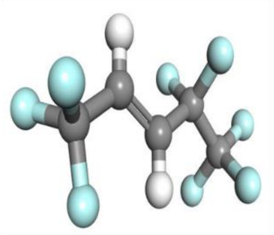

1,1,1,4,4,5,5,5-octafluoropent-2-ene

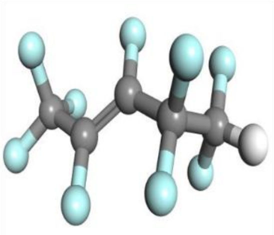

(E)-1,1,1,2,3,4,4,5,5-nonafluoropent-2-ene

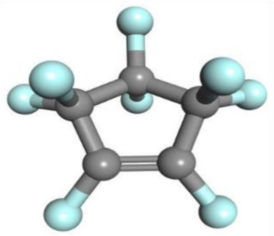

1,2,3,3,4,4,5,5-octafluorocyclopentene

Figure S39. 22th set of organic compounds for validation of computational protocol. Chemical structures of 22th set of organic compounds introduced for the validation process of the DFT-based prediction protocol of the dielectric strength in Figure 5.

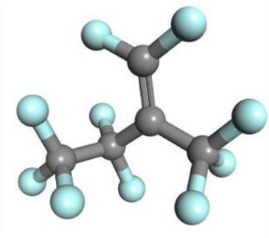

1,1,3,3,4,4,4-heptafluoro-2-(trifluoromethyl)but-1-ene

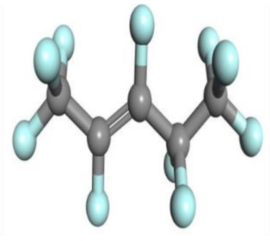

(E)-1,1,1,2,3,4,4,5,5,5-decafluoropent-2-ene

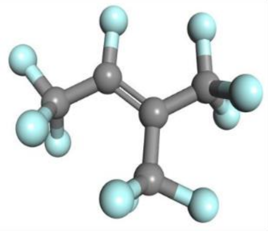

1,1,1,2,4,4,4-heptafluoro-3-(trifluoromethyl)but-2-ene

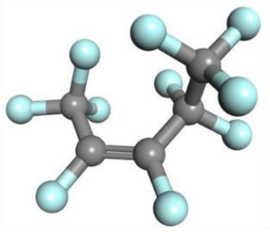

(Z)-1,1,1,2,3,4,4,5,5,5-decafluoropent-2-ene

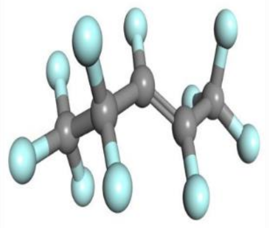

1,1,1,2,3,4,4,5,5,5-decafluoropent-2-ene

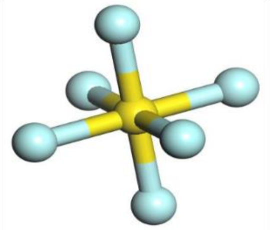

Sulfur hexafluoride

Figure S40. 23th set of organic compounds for validation of computational protocol. Chemical structures of 23th set of organic compounds introduced for the validation process of the DFT-based prediction protocol of the dielectric strength in Figure 5.
